# The ORCA2 transcription factor plays a key role in regulation of the terpenoid indole alkaloid pathway

**DOI:** 10.1186/1471-2229-13-155

**Published:** 2013-10-08

**Authors:** Chun Yao Li, Alex L Leopold, Guy W Sander, Jacqueline V Shanks, Le Zhao, Susan I Gibson

**Affiliations:** 1 Department of Plant Biology, University of Minnesota Twin Cities, Saint Paul, MN 55108, USA; 2 Department of Chemical Engineering, University of Minnesota Duluth, Duluth, MN 55812, USA; 3 Department of Chemical and Biological Engineering, Iowa State University, Ames, IA 50011, USA

**Keywords:** Terpenoid indole alkaloids, ORCA2, *Catharanthus roseus*, Hairy root cultures

## Abstract

**Background:**

The terpenoid indole alkaloid (TIA) pathway leads to the production of pharmaceutically important drugs, such as the anticancer compounds vinblastine and vincristine. Unfortunately, these drugs are produced in trace amounts, causing them to be very costly. To increase production of these drugs, an improved understanding of the TIA regulatory pathway is needed. Towards this end, transgenic *Catharanthus roseus* hairy roots that overexpress the *ORCA2* TIA transcriptional activator were generated and characterized.

**Results:**

Transcriptional profiling experiments revealed that overexpression of *ORCA2* results in altered expression of key genes from the indole and terpenoid pathways, which produce precursors for the TIA pathway, and from the TIA pathway itself. In addition, metabolite-profiling experiments revealed that overexpression of *ORCA2* significantly affects the levels of several TIA metabolites. *ORCA2* overexpression also causes significant increases in transcript levels of several TIA regulators, including TIA transcriptional repressors.

**Conclusions:**

Results presented here indicate that ORCA2 plays a critical role in regulation of TIA metabolism. ORCA2 regulates expression of key genes from both feeder pathways, as well as the genes (*STR* and *SGD*) encoding the enzymes that catalyze the first two steps in TIA biosynthesis. ORCA2 may play an especially important role in regulation of the downstream branches of the TIA pathway, as it regulates four out of five genes characterized from this part of the pathway. Regulation of TIA transcriptional repressors by ORCA2 may provide a mechanism whereby increases in TIA metabolite levels in response to external stimuli are transient and limited in magnitude.

## Background

The plant *Catharanthus roseus* (L.) G. Don (Madagascar periwinkle) produces a large number of terpenoid indole alkaloids (TIAs), some of which are of substantial pharmacological interest. Vinblastine and vincristine have been used as chemotherapeutics in the treatment of lymphoma and leukemia [1]. Ajmalicine and serpentine are sometimes used as anti-hypertensive agents. Most of these alkaloids are produced in extremely low amounts *in planta*, limiting the usage of these chemicals. Substantial efforts to use chemical syntheses, *in vitro* cell cultures or bacterial cells for large-scale production of these alkaloids have proven ineffective [2,3]. One of the difficulties in developing methods for large-scale production of TIAs is the complexity of both the TIA biosynthetic pathway and of the regulatory pathways governing TIA production. Despite the complexity of these pathways, significant progress in our understanding of the biochemistry and regulation of the TIA pathway in *C. roseus* has been made in recent years. In particular, many of the genes coding for the TIA biosynthetic enzymes and TIA transcriptional activators and repressors have been identified [4-9].

**Figure 1:**
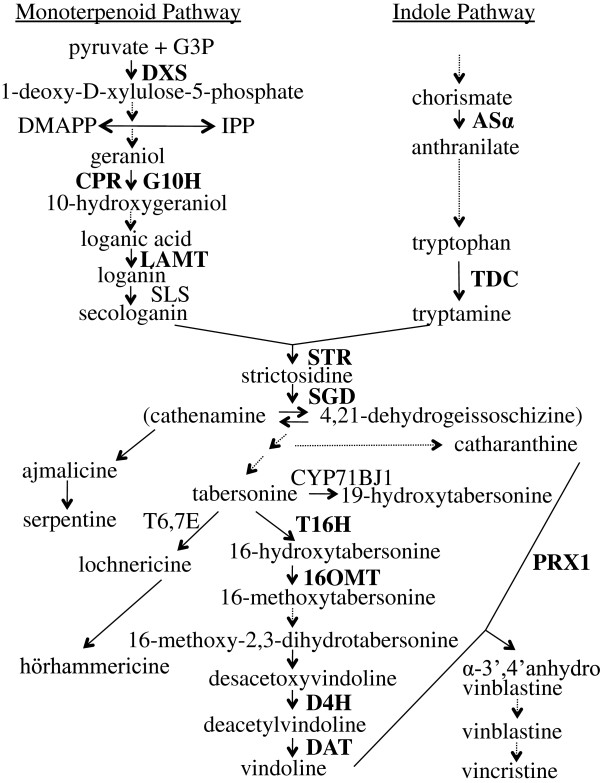
**TIA biosynthesis in *C. roseus*.** Enzyme abbreviations are written in capital letters next to the arrow indicating the reaction catalyzed by each enzyme. The metabolites resulting from different enzymatic conversions are indicated by the appropriate arrows. Solid arrows represent single enzymatic conversions, whereas dashed arrows indicate multiple enzymatic reactions. The genes that were included for analysis of expression are marked in bold. 16OMT, 16-hydroxytabersonine-*O*-methyl-transferase; AS, anthranilate synthase; CPR, cytochrome P450 reductase; CYP71BJ1, CYP71 cytochrome P450 hydroxylase; D4H, desacetoxyvindoline 4-hydroxylase; DAT, deacetylvindoline 4-*O*-acetyltransferase; DMAPP, dimethylallyl pyrophosphate; DXS, 1-deoxy-D-xylulose-5-phosphate synthase; G3P, glyceraldehyde 3-phosphate; G10H, geraniol 10-hydroxylase; IPP, isopentenyl diphosphate; LAMT, loganic acid *O*-methyltransferase; PRX1, vacuolar class III peroxidase; SGD, strictosidine glucosidase; SLS, secologanin synthase; STR, strictosidine synthase; T6,7E, tabersonine 6,7-epoxidase; T16H, tabersonine 16-hydroxylase; TDC, tryptophan decarboxylase.

TIA biosynthesis is a tightly coordinated process involving at least 35 intermediates, 30 biosynthetic enzymes and several intra and inter cellular compartments [3,10,11]. TIA biosynthesis in *C. roseus* starts with the condensation of tryptamine, which is derived from the indole pathway, and secologanin, which is derived from the monoterpenoid pathway, to form strictosidine (Figure 1). This reaction is catalyzed by strictosidine synthase (STR). Strictosidine is then deglucosylated by strictosidine β-D-glucosidase (SGD) to form cathenamine and an equilibrium between cathenamine and 4,21-dehydrogeissoschizine is established [12]. Different branches of the TIA pathway then lead to the production of numerous TIAs, including ajmalicine and serpentine, lochnericine and hörhammericine, and catharanthine and vindoline. Vindoline and catharanthine are the precursors of vinblastine and vincristine. The genes encoding the enzymes catalyzing at least four of the reactions in the six-step pathway leading to production of vindoline from tabersonine have been cloned and characterized. These genes include *tabersonine 16-hydroxylase* (*T16H*) [13], *16-hydroxytabersonine-16-*O*-methyltransferase* (*16OMT*) [14], *desacetoxyvindoline 4-hydroxylase* (*D4H*) [15], and *deacetylvindoline acetyltransferase* (*DAT*) [16]. An enzyme with anhydrovinblastine synthase activity that catalyzes the synthesis of α-3′, 4′-anhydrovinblastine from vindoline and catharanthine has also been identified and characterized as the major class III peroxidase (PRX1) in *C. roseus* leaves [17].

TIA biosynthesis is a highly regulated process that involves a number of transcriptional activators and repressors. To date, seven putative activators (ORCA2, ORCA3, CrBPF1, CrMYC1, CrMYC2, CrWRKY1 and CrWRKY2) and five putative repressors (ZCT1, ZCT2, ZCT3, GBF1 and GBF2) have been implicated as regulators of the TIA pathway. However, very few studies have been done on any of these regulators, with the exception of ORCA3 [7-9,18-24]. Both ORCA2 (Octadecanoid-Responsive Catharanthus AP2-domain protein 2) [18] and ORCA3 [22] are AP2-domain transcription factors that are proposed to activate *STR* expression by binding to the jasmonate and elicitor-responsive element (JERE) in the *STR* promoter [18,22]. CrBPF1 is also proposed to activate *STR* transcription by binding to a separate element in the *STR* promoter [24]. CrMYC1 [23] and CrMYC2 [7] are basic helix-loop-helix transcription factors. CrMYC2 has been shown to act upstream of ORCA2 and ORCA3, activating their transcription [7]. CrWRKY1 and CrWRKY2 are jasmonate responsive WRKY transcription factors that positively regulate expression of several genes involved in TIA biosynthesis [8,9]. Overexpression of *CrWRKY1* also leads to increased transcript levels of the TIA transcriptional repressors *ZCT1*, *ZCT2* and *ZCT3* and decreased transcript levels of the TIA transcriptional activators *ORCA2, ORCA3* and *CrMYC2*[9]. In contrast, overexpression of *CrWRKY2* leads to increased expression of both specific TIA transcriptional activators (*ORCA2*, *ORCA3* and *CrWRKY1*) and repressors (*ZCT1* and *ZCT3*) [8]. The three zinc finger proteins, ZCT1, ZCT2, and ZCT3, were found to bind specifically to the *tryptophan decarboxylase* (*TDC)* and *STR* promoters *in vitro,* inhibiting their activities. In addition, the ZCT proteins repress the activation of the *STR* promoter by the ORCAs [19]. Two G-box-binding factors, GBF1 and GBF2, were found to repress *STR* transcription by binding to the G-box sites in the *STR* promoter region [20].

The identification of multiple transcriptional activators and repressors for the TIA pathway suggests that regulation of this pathway is a complex process. However, to date there have been very few published studies describing in depth characterization of TIA regulators other than ORCA3. ORCA3 has been the focus of several studies in recent years. The results of these studies indicate that ORCA3 acts as a positive regulator of many TIA biosynthetic genes [5,19,21,22,25-30]. For these studies *ORCA3* was overexpressed in either suspension cells or hairy root cultures. In *C. roseus* cell cultures, overexpression of *ORCA3* increases the transcript levels of *TDC*, *STR*, *cytochrome P450 reductase* (*CPR*), *1-deoxy-D-xylulose 5-phosphate synthase* (*DXS*), *anthranilate synthase α subunit* (*ASα*) and *D4H*. In contrast, overexpression of *ORCA3* in *C. roseus* cell suspension cultures has no significant effects on transcript levels of *geraniol 10-hydroxylase* (*G10H*), *SGD* or *DAT*[21]. In *C. roseus* hairy root cultures, overexpression of *ORCA3* induces expression of *ASα*, *DXS*, *secologanin synthase* (*SLS*), *STR* and *ZCT1, ZCT2* and *ZCT3,* but represses *SGD* expression. The transcript levels of *TDC*, *CPR*, *G10H*, *GBF1*, *GBF2* and *ORCA2* were not significantly affected by *ORCA3* overexpression [27].

Although several studies have characterized the role of ORCA3 in regulating the TIA pathway, little work has been done to characterize the role of the related ORCA2 transcriptional activator. Recently Liu et al. reported the generation of *C. roseus* transgenic hairy root lines that exhibit constitutive overexpression of *ORCA2* and stated that catharanthine and vindoline concentrations increased in these lines, but only HPLC and an authentic standard were used for the identification of vindoline [31]. Vinblastine levels in these lines were below detection limits and other TIA metabolites were not assayed as part of this study. The expression levels of TIA biosynthetic genes and of other TIA regulatory genes were also not characterized by Liu and colleagues, leaving the role of ORCA2 in regulating TIA metabolism unknown.

Here we describe the generation, metabolic and molecular characterization of a transgenic *C. roseus* hairy root line that expresses *ORCA2* under the control of an ethanol-inducible promoter. The transcript levels of a total of 22 TIA biosynthetic and regulatory genes were tracked over a period of 72 h following induction of *ORCA2* overexpression. The levels of seventeen TIA and related metabolites were also investigated over the same time period, with thirteen of those metabolites being present at detectable levels in at least some of the samples analyzed. The results of these experiments indicate that ORCA2 plays an important role in regulating the TIA pathway, particularly the downstream portions of this pathway. Based on the results of these experiments, a model for regulation of the TIA pathway by ORCA2 and other TIA transcriptional regulators is presented.

## Results

### Generation of *C. roseus* transgenic hairy root lines expressing *ORCA2* under the control of an ethanol-inducible promoter

To date, only a few studies have characterized ORCA2 [18,31] and no published studies have determined which TIA genes, other than *STR*, are regulated by ORCA2 or how *ORCA2* overexpression affects the levels of a broad group of TIA metabolites. As a result, determining the role of ORCA2 in regulating the TIA pathway has not been possible. To address this deficiency, transgenic hairy root lines that overexpress *ORCA2* under the control of an ethanol-inducible promoter were generated. The ethanol-inducible system offers significant advantages over constitutive expression systems in that the ethanol-inducible system allows the timing and level of transgene expression to be controlled [32]. As a result, studies on the transient effects of transgene expression are made possible. In addition, the potentially deleterious effects of constitutive overexpression of transgenes on tissue growth and development may be mitigated.

**Figure 2:**
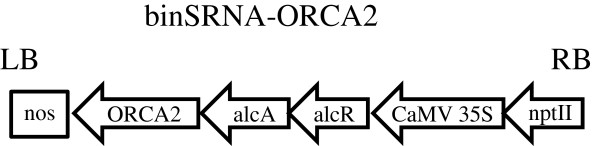
**Schematic of binSRNA-ORCA2 overexpression construct used for *C. roseus *transformation.** The CaMV 35S promoter directs transcription of the *alcR* gene to produce the regulatory protein, AlcR. In the presence of its coinducer, AlcR binds to specific regions within the *alcA* promoter region and induces transcription of downstream genes [34]. As plants produce the AlcR coinducer, acetaldehyde [35], from ethanol, this construct thus allows ethanol-inducible expression of *ORCA2*. The *npt11* gene codes for kanamycin resistance. The transcription termination sequence from the *nos* gene is present downstream of *ORCA2*. alcA, *alcA* promoter sequence; alcR, *alcR* coding region; CaMV 35S, CaMV 35S promoter sequence; LB, T-DNA left border sequence; nos, nopaline synthase transcription termination sequence; ORCA2, *ORCA2* cDNA coding region; RB, T-DNA right border sequence.

**Figure 3:**
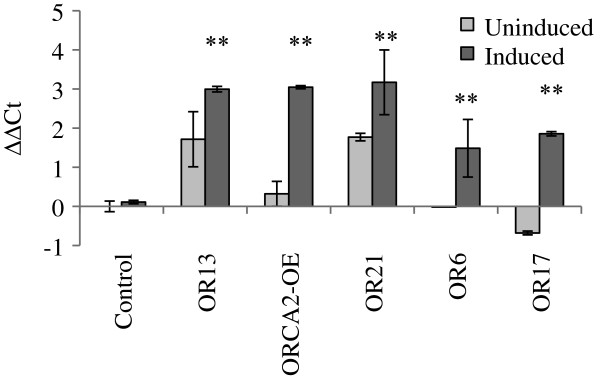
**Relative *ORCA2 *transcript levels in five ORCA2 transgenic hairy root lines.***ORCA2* transcript levels were assayed in five transgenic hairy root lines transformed with the binSRNA-ORCA2 and pPZPROL constructs (OR6, OR13, OR17, OR21 and ORCA2-OE) and in a control line carrying only the pPZPROL construct (Control). *ORCA2* transcript levels were measured in these lines under induced and uninduced conditions, 10 h after induction with 0.5% ethanol. Relative mRNA levels are presented as ∆∆CT. ∆∆CT = ∆CT_uninduced control line_ - ∆CT_other line_, where ∆CT = CT_*ORCA2*_ - CT_*EF1*_ and “other line” is the induced control line or uninduced or induced cultures of one of the binSRNA-ORCA2 containing lines. Positive ∆∆CT values indicate that *ORCA2* transcript levels are higher in the indicated hairy root line grown under the indicated conditions than in the uninduced control line. Conversely, negative ∆∆CT values indicate that *ORCA2* transcript levels are lower in the indicated hairy root line grown under the indicated conditions than in the uninduced control line. Results are the average ∆∆CT value of three biological replicates, with two technical replicates for each biological replicate. Error bars indicate standard deviations. ** indicates that *ORCA2* transcript levels in the induced versus uninduced cultures of a hairy root line carrying the binSRNA-ORCA2 construct differed by *p* ≤ 0.01, according to Student’s t-test.

To generate transgenic hairy roots, approximately 6-week-old *C. roseus* seedlings were co-transformed using a mixture of *Agrobacterium tumefaciens* GV3101 carrying the binSRNA-ORCA2 construct (Figure 2) and GV3101 carrying the pPZPROL plasmid. The pPZPROL plasmid was previously engineered to carry the *rolA*, *rolB* and *rolC* genes that are sufficient to induce hairy root production [33]. A total of 27 hairy root lines were screened for genomic integration of *ORCA2* by assaying for kanamycin resistance and using PCR to identify a DNA fragment spanning the *ORCA2* gene and *alcA* promoter. Five of the 27 hairy root lines were found to carry *ORCA2* under the control of the *alcA* promoter. These positive lines were transferred to liquid culture for adaptation. Following adaptation, the lines were screened for ethanol-inducible overexpression of *ORCA2*. Figure 3 shows *ORCA2* relative transcript levels from the five ORCA2 transgenic hairy root lines. The results indicate that the transgenic lines express *ORCA2* transcripts at approximately three to 10 times higher levels than the control line 10 h after induction by 0.5% (v/v) ethanol. As the ORCA2-OE line exhibited one of the highest levels of *ORCA2* induction and adapted well to liquid culture, this line was retained for further study. A control line was also generated by transforming *C. roseus* seedlings with GV3101 cells carrying the pPZPROL construct alone.

**Figure 4:**
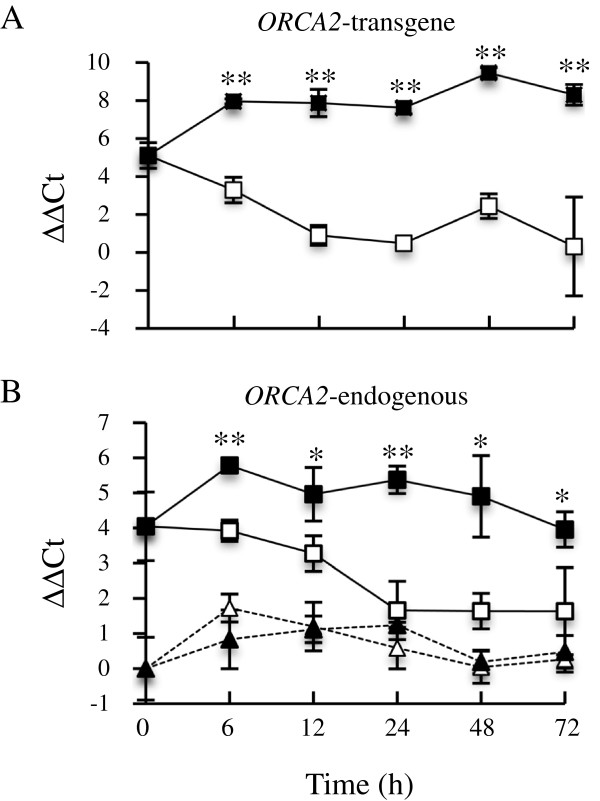
**Time course analysis of *ORCA2 *expression.***ORCA2* transcripts generated from the endogenous *ORCA2* gene and from the *ORCA2* transgene were measured separately by qPCR, using primer pairs specific for each gene. The PCR primer pair used to analyze *ORCA2* transgene transcripts, as expected, gives only a very low background signal for the control line (which lacks the *ORCA2* transgene, data not shown). The lines analyzed are: uninduced ORCA2-OE line (□, solid line), 0.02% ethanol-induced ORCA2-OE line (■, solid line), uninduced control line (∆, dashed line) and 0.02% ethanol-induced control line (▲, dashed line). **A)** Relative *ORCA2* transgene transcript levels are presented as ∆∆CT. Note that *ORCA2* transgene transcript levels were normalized versus *ORCA2* endogenous gene transcript levels in the uninduced control line at 0 h rather than against *ORCA2* transgene transcript levels in the uninduced control line at 0 h because the control line does not carry the *ORCA2* transgene. **B)** Relative *ORCA2* endogenous gene transcript levels are presented as ∆∆CT. Positive ∆∆CT values indicate that *ORCA2* endogenous gene transcript levels are higher in the indicated hairy root line grown for the indicated time under the indicated conditions than in the uninduced control line at 0 h. Negative ∆∆CT values indicate the reverse. Results are the average ∆∆CT value of three biological replicates, with two technical replicates for each biological replicate. Error bars indicate standard deviations. *ORCA2* transcript levels in the induced versus uninduced ORCA2-OE cultures differed at the same time point with: * = p ≤ 0.05, ** = p ≤ 0.01 according to Student’s t-test. Student’s t-test results for the induced versus uninduced cultures of the control line are not depicted.

To determine if ethanol-induced overexpression of *ORCA2* in the ORCA2-OE line is maintained for a substantial period of time, a 72-h time course was performed. ORCA2 transcripts generated from the *ORCA2* transgene and by the endogenous *ORCA2* gene were measured separately in this experiment. An ethanol concentration of 0.02% was used for this experiment after this concentration of ethanol was found to be sufficient to cause increases in *ORCA2* transcript levels similar to those caused by the 0.5% ethanol used in earlier experiments (data not shown). Figure 4A shows that *ORCA2* transgene transcript levels increase dramatically after adding ethanol to ORCA2-OE root cultures, rising over seven fold within 6 h. *ORCA2* transgene transcript levels remained high in the induced cultures over the 72-h course of the experiment. In contrast, *ORCA2* transgene transcript levels decrease in uninduced ORCA2-OE root cultures upon transfer to fresh media at the 0-h time point. This decrease in *ORCA2* transgene transcript levels is likely due to the fact that plant cultures grown in liquid typically produce small amounts of ethanol. Thus, the 35-d old cultures from which the 0-h time point samples were collected contained small amounts of ethanol, which likely caused a modest induction of *ORCA2* transgene expression. Transferring additional 35-d old cultures to fresh media with no added ethanol at the 0-h time point thus caused a slight decrease in ethanol concentration and, consequently, in *ORCA2* transgene expression in those cultures. As a result, *ORCA2* transgene transcript levels are approximately 100-fold higher in the induced than in the uninduced ORCA2-OE cultures within approximately 12 h after induction.

The levels of *ORCA2* transcripts generated from the endogenous *ORCA2* gene increase approximately three fold after addition of ethanol to the ORCA2-OE cultures and then decline back to starting levels by the end of the 72-h time course (Figure 4B). In contrast, *ORCA2* endogenous gene transcript levels decline in the ORCA2-OE cultures transferred to fresh media with no added ethanol. These results suggest that ORCA2, directly or indirectly, induces expression of the *ORCA2* endogenous gene, as cultures with high levels of expression of the *ORCA2* transgene express the endogenous *ORCA2* gene at higher levels than cultures with low levels of expression of the *ORCA2* transgene. Consistent with this hypothesis, *ORCA2* endogenous gene transcript levels are significantly higher in ORCA2-OE cultures than in control cultures at the 0 h time point, as *ORCA2* transgene expression is modestly induced in these ORCA2-OE cultures by plant-produced ethanol. *ORCA2* endogenous gene transcript levels are not significantly different in uninduced versus induced cultures of the control line.

### Effects of *ORCA2* overexpression on the indole and terpenoid pathways

To determine if ORCA2 plays an important role in regulation of the TIA pathway, transcript levels of 22 genes encoding key enzymes and regulators of the TIA and related pathways were analyzed in the ORCA2-OE and control hairy root lines. The genes analyzed include *ASα* and *TDC*, two key genes from the indole pathway that produces tryptamine; *DXS*, *CPR*, *G10H* and *loganic acid* O*-methyltransferase (LAMT)* from the monoterpenoid pathway which leads to the formation of secologanin; and *STR*, *SGD*, *T16H*, *16OMT*, *D4H*, *DAT* and *PRX1* from the TIA pathway which catalyzes the condensation of tryptamine and secologanin and ultimately leads to the production of vinblastine and vincristine. Transcript levels of four transcriptional activators (*ORCA2, ORCA3*, *CrBPF1* and *CrMYC2*) and five repressors (*ZCT1, ZCT2, ZCT3, GBF1* and *GBF2*) that regulate the TIA pathway were also analyzed. To analyze the downstream effects of *ORCA2* overexpression, the levels of 17 TIA and related metabolites were also investigated, with 13 of those metabolites found to be present at detectable levels in at least some of the samples analyzed. Both transcript and metabolite levels were tracked over a 72-h period following ethanol induction of *ORCA2* overexpression, to allow analysis of both transient and relatively prolonged effects.

**Figure 5:**
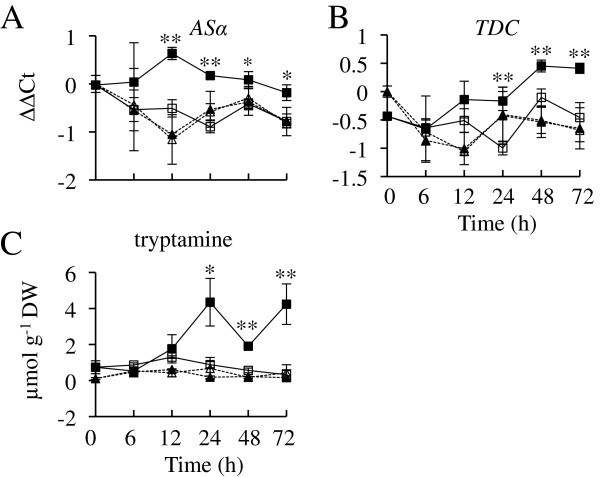
**Time course analysis of *ASα *and *TDC *transcript and tryptamine metabolite levels.** Tryptophan levels were below detection thresholds. Results shown are the: uninduced ORCA2-OE line (□, solid line), 0.02% ethanol-induced ORCA2-OE line (■, solid line), uninduced control line (∆, dashed line) and 0.02% ethanol-induced control line (▲, dashed line). **A)** Relative *AS*α transcript levels are presented as ∆∆CT. **B)** Relative *TDC* transcript levels are presented as ∆∆CT. Positive ∆∆CT values indicate that transcript levels for the indicated gene are higher in the indicated hairy root line grown for the indicated time under the indicated conditions than in the uninduced control line at 0 h. Conversely, negative ∆∆CT values indicate that transcript levels are lower for the indicated gene in the indicated hairy root line grown for the indicated time under the indicated conditions than in the uninduced control line at 0 h. Results for transcript levels are the average ∆∆CT values of three biological replicates, with two technical replicates for each biological replicate. **C)** Tryptamine levels are depicted. Results for metabolite levels are the averages of three biological replicates. Error bars indicate standard deviations. Transcript levels of the indicated gene in the induced versus uninduced ORCA2-OE cultures differed at the same time point with: * = p ≤ 0.05, ** = p ≤ 0.01 according to Student’s t-test. Similarly, metabolite levels differed with: * = p ≤ 0.1, ** = p ≤ 0.05 according to Student’s t-test. Student’s t-test results for the induced versus uninduced cultures of the control line are not depicted.

To characterize the effects of *ORCA2* overexpression on the indole pathway, *ASα* and *TDC* transcript levels were analyzed. *ASα* encodes the *α* subunit of anthranilate synthase, which catalyzes the first committed step in the synthesis of tryptophan, the formation of chorismate from anthranilate. *TDC* encodes tryptophan decarboxylase, which catalyzes the conversion of tryptophan to tryptamine. Both *ASα* and *TDC* transcript levels are significantly higher in the ORCA2-OE line after ethanol induction than in the uninduced ORCA2-OE cultures or in the induced or uninduced cultures of the control line (Figure 5A and B). *ASα* expression in the ORCA2-OE line increases within 12 h of the start of *ORCA2* induction and then decreases slightly, but remains significantly higher than in the uninduced ORCA2-OE cultures or in the induced or uninduced cultures of the control line. *TDC* transcript levels increase within 24 h and increase further within 48 h after the start of *ORCA2* induction. The levels of tryptamine, the product of the indole pathway that is combined with the terpenoid secologanin to form the first TIA, strictosidine, were also analyzed in samples from the same hairy root cultures. Tryptamine levels increase significantly within 24 h of induction and remain increased through 72 h. Tryptamine levels remain unchanged in the uninduced ORCA2-OE cultures and the induced and uninduced cultures of the control line (Figure 5C). Levels of tryptophan were below detection limits.

**Figure 6:**
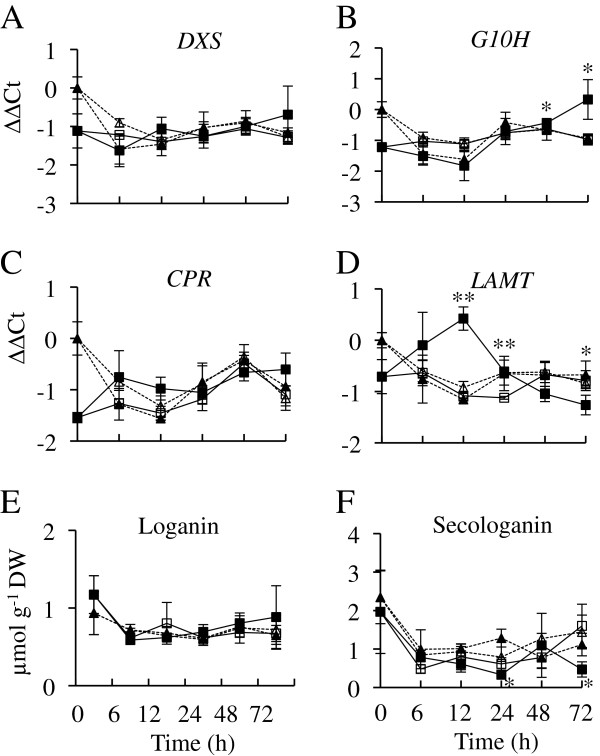
**Time course analysis of transcript and metabolite levels from the terpenoid pathway.** Results shown are the: uninduced ORCA2-OE line (□, solid line), 0.02% ethanol-induced ORCA2-OE line (■, solid line), uninduced control line (∆, dashed line) and 0.02% ethanol-induced control line (▲, dashed line). The relative mRNA levels are presented as ∆∆CT. Results depicted are as follows: **A)***DXS* transcript levels, **B)***G10H* transcript levels, **C)***CPR* transcript levels, **D)***LAMT* transcript levels, **E)** loganin levels and **F)** secologanin levels. Results for transcript levels are the average ∆∆CT values of three biological replicates, with two technical replicates for each biological replicate. Results for metabolite levels are the averages of three biological replicates. Error bars indicate standard deviations. Transcript levels of the indicated gene in the induced versus uninduced ORCA2-OE cultures differed at the same time point with: * = p ≤ 0.05, ** = p ≤ 0.01 according to a Student’s t-test. Similarly, metabolite levels differed with: * = p ≤ 0.1, ** = p ≤ 0.05 according to Student’s t-test. Student’s t-test results for the induced versus uninduced cultures of the control line are not depicted.

To characterize the effects of *ORCA2* overexpression on the terpenoid pathway, the transcript levels of *DXS*, *G10H*, *CPR* and *LAMT* were analyzed (Figure 6). Transcript levels of *DXS* and *CPR* are not significantly different in the induced ORCA2-OE line than in the uninduced ORCA2-OE line (Figure 6A and C). In contrast, *G10H* transcript levels in the ORCA2-OE line increase within 48 h after the start of *ORCA2* induction. Expression of *LAMT* increases sharply in the ORCA2-OE line after induction, but then decreases within less than 24 h, reaching levels that are lower than those seen in uninduced ORCA2-OE cultures within 72 h (Figure 6D). Addition of ethanol to the “induced” control cultures had little affect on gene expression, indicating that differences in ethanol concentration *per se* are not responsible for the differences in gene expression observed for the induced versus uninduced ORCA2-OE cultures. The differences in gene expression observed between the control and ORCA2-OE cultures at the 0-h time point may be due to the fact that different clonal root cultures have somewhat different growth characteristics. Alternatively, some of these differences may be due to the fact that the 35-d old ORCA2-OE cultures from which the 0-h time point samples were collected likely contained small amounts of root-produced ethanol, which led to a modest induction of *ORCA2* transgene expression.

The effects of *ORCA2* overexpression on loganin and secologanin levels were also determined. Loganin is the precursor for secologanin, which is one of the precursors for formation of the first TIA, strictosidine. Loganin levels in induced and uninduced ORCA2-OE cultures are similar (Figure 6E). In contrast, secologanin levels are somewhat variable in the ORCA2-OE line. Secologanin levels are significantly decreased in induced ORCA2-OE cultures relative to uninduced ORCA2-OE cultures at 24 and 72 h after induction, but are not significantly different at the other time points assayed (Figure 6F).

### Effects of *ORCA2* overexpression on the TIA pathway

**Figure 7:**
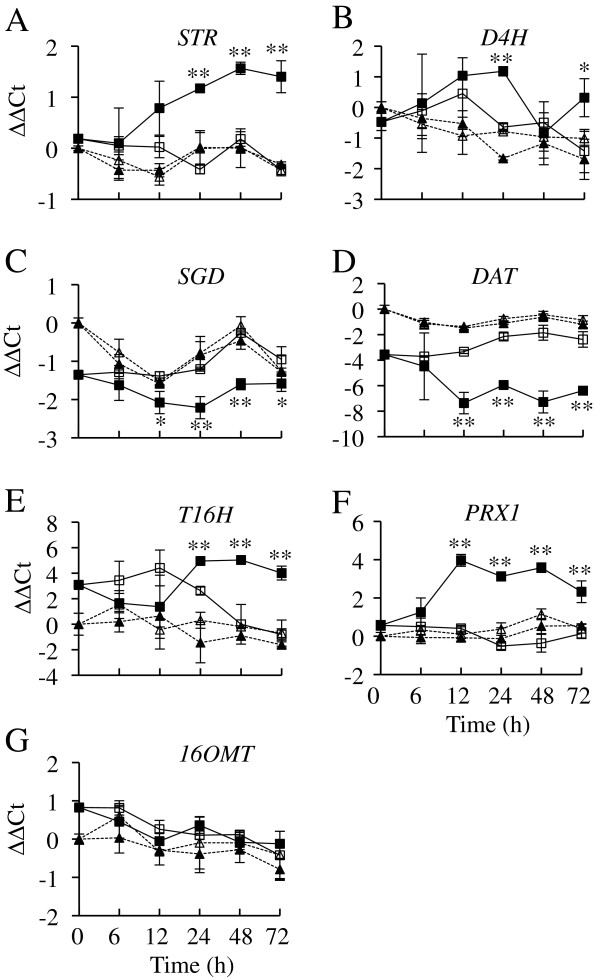
**Time course analysis of transcript levels of TIA biosynthetic genes.** Results shown are the: uninduced ORCA2-OE line (□, solid line), 0.02% ethanol-induced ORCA2-OE line (■, solid line), ethanol uninduced control line (∆, dashed line) and 0.02% ethanol-induced control line (▲, dashed line). The relative mRNA levels are presented as ∆∆CT. Results depicted are as follows: **A)***STR* transcript levels, **B)***D4H* transcript levels, **C)***SGD* transcript levels, **D)***DAT* transcript levels, **E)***T16H* transcript levels, **F)***PRX1* transcript levels and **G)***16OMT* transcript levels. Results are the average ∆∆CT values of three biological replicates, with two technical replicates for each biological replicate. Error bars indicate standard deviations. Transcript levels of the indicated gene in the induced versus uninduced ORCA2-OE cultures differed at the same time point with: * = p ≤ 0.05, ** = p ≤ 0.01 according to Student’s t-test. Student’s t-test results for the induced versus uninduced cultures of the control line are not depicted.

The first step in TIA synthesis, the condensation of tryptamine and secologanin to form strictosidine, is catalyzed by STR. SGD then uses strictosidine as a substrate to form cathenamine, which serves as a substrate for the formation of several TIAs via different branches of the TIA pathway. To test if ORCA2 plays an important role in regulating these processes, transcript levels for *STR, SGD, T16H, 16OMT, D4H, DAT* and *PRX1* were analyzed in the ORCA2-OE and control hairy root lines for a 72-h period following *ORCA2* induction (Figure 7). The results of these experiments indicate that transcript levels of six of the seven genes tested are significantly affected by *ORCA2* overexpression. *STR*, *T16H* and *PRX1* are strikingly induced by *ORCA2* overexpression, although with somewhat different timing and magnitudes. *STR* transcript levels increase significantly in the ORCA2-OE cultures within 24 h of the start of *ORCA2* induction, reaching a maximum of approximately three times higher levels at the 48-h time point than in the ORCA2-OE cultures at the zero time point. *T16H* transcript levels also increase significantly within 24 h of the start of induction, reaching levels by the 48 h time point that are 20 to 30 times higher in the induced ORCA2-OE cultures than in the uninduced ORCA2-OE cultures at the same time point. *PRX1* transcript levels increase approximately 16 fold in the ORCA2-OE cultures within 12 h after the start of induction and then decline slightly. *D4H* transcript levels are significantly higher in the induced than in the uninduced ORCA2-OE cultures at 24 and 72 h after the start of induction, but are only increased by approximately two to four fold. Interestingly, transcript levels of both *SGD* and *DAT* decrease in response to *ORCA2* overexpression. The timing of the declines in *SGD* and *DAT* transcript levels are similar, with both genes exhibiting significantly lower transcript levels in the induced versus uninduced ORCA2-OE cultures within 12 h of the start of induction. However, the magnitudes of the changes in transcript levels are different, as *SGD* transcript levels are only approximately two fold lower in the induced versus uninduced ORCA2-OE cultures, whereas *DAT* transcript levels are approximately 15 to 40 fold lower in the induced versus uninduced ORCA2-OE cultures. Of the seven TIA biosynthetic genes characterized, only *16OMT* does not show significant differences in expression levels between uninduced and induced ORCA2-OE hairy root cultures. The differences in expression of some of the genes between the control and ORCA2-OE cultures at the 0-h time point may be due to different clonal root cultures having somewhat different growth characteristics and/or to the fact that the ORCA2-OE cultures likely expressed the *ORCA2* transgene at modest levels at the 0-h time point due to the presence of small amounts of root-produced ethanol in the 35-d old cultures from which those samples were collected.

**Figure 8:**
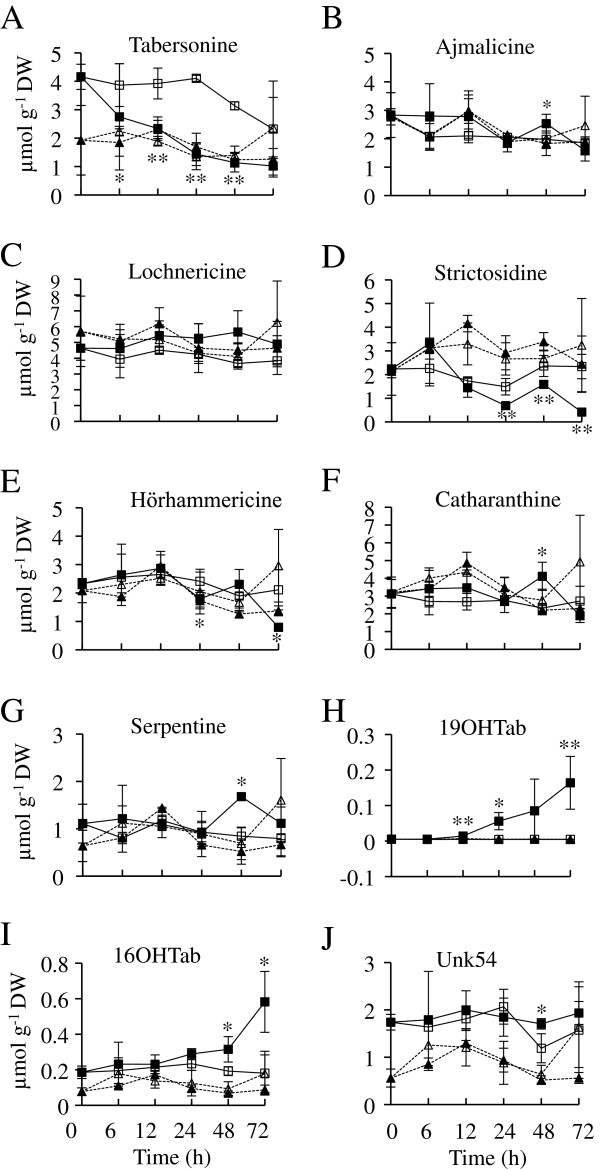
**Time course analysis of TIA metabolite levels.** Levels of vindoline, vinblastine and vincristine were below detection thresholds. Unk54 represents a tabersonine-like compound. Results shown are the: uninduced ORCA2-OE line (□, solid line), 0.02% ethanol-induced ORCA2-OE line (■, solid line), uninduced control line (∆, dashed line) and 0.02% ethanol-induced control line (▲, dashed line). Levels of the following metabolites are presented: **A)** tabersonine, **B)** ajmalicine, **C)** lochnericine, **D)** strictosidine, **E)** hörhammericine, **F)** catharanthine, **G)** serpentine, **H)** 19OHTab, **I)** 16OHTab and **J)** Unk54. Results are the average values of three biological replicates. Error bars indicate standard deviations. Levels of the indicated metabolite in the induced versus uninduced ORCA2-OE cultures differed at the same time point with: * = p ≤ 0.1, ** = p ≤ 0.05 according to Student’s t-test. Student’s t-test results for the induced versus uninduced cultures of the control line are not depicted.

As *ORCA2* overexpression causes significant alterations in the transcript levels of a high percentage of the TIA biosynthetic genes tested, it was of interest to determine if *ORCA2* overexpression also alters TIA levels. To address this issue, the levels of 12 TIAs from different branches of the TIA pathway were investigated in aliquots of the same tissue samples used to analyze transcript levels. Nine of these TIAs were present at detectable levels, whereas the levels of the other three TIAs (vindoline, vinblastine and vincristine) were below detection thresholds. In addition, a tabersonine-like compound, designated Unk54, was detected in the samples (Figure 8). The levels of strictosidine, the first TIA to be formed from secologanin and tryptamine, are significantly reduced in the induced versus uninduced ORCA2-OE cultures within 24 h after the start of induction and remain reduced throughout the remainder of the 72-h time course. Ajmalicine and serpentine are formed via the same branch of the TIA pathway (Figure 1). Both ajmalicine and serpentine levels exhibit modestly significant increases in the induced versus uninduced ORCA2-OE cultures 48 h after the start of induction, but are not significantly different at the other time points assayed. Catharanthine, the precursor for vinblastine production, also exhibits modestly significantly higher levels 48 h after induction, but not at the other time points assayed. The levels of tabersonine, which is the starting material for multiple branches of the TIA pathway, are significantly reduced in induced versus uninduced ORCA-OE cultures within 6 h of the start of *ORCA2* induction. Tabersonine is converted via one branch of the TIA pathway to lochnericine and hörhammericine. Lochnericine levels are not significantly altered by *ORCA2* overexpression, but hörhammericine levels are significantly reduced in the induced versus uninduced ORCA2-OE cultures at 24 and 72 h after the start of induction. Tabersonine is converted via a different branch of the TIA pathway to 16-hydroxytabersonine (16OHTab). 16OHTab levels exhibit modestly significant increases in the induced versus uninduced ORCA2-OE cultures at 48 and 72 h after the start of *ORCA2* induction. Tabersonine is also converted to 19-hydroxytabersonine (19OHTab). 19OHTab levels increase within 12 h of the start of ORCA2 induction and remain elevated for at least 72 h. Levels of vindoline, the end metabolite of this branch of the TIA pathway and precursor to vinblastine production, are below detection limits. An un-identified metabolite, designated Unk54, was also detected in the tissue extracts. Based on its UV absorption spectra between 200 and 400 nm (data not shown), Unk54 is a tabersonine-like compound. Unk54 levels exhibit modestly significant increases in the induced versus uninduced ORCA2-OE cultures at 48 h after the start of induction, but not at the other time points assayed. The levels of the pharmaceutically important TIAs vinblastine and vincristine, formed by combining catharanthine and vindoline, were below detection limits.

### Effects of *ORCA2* overexpression on regulators of the TIA pathway

**Figure 9:**
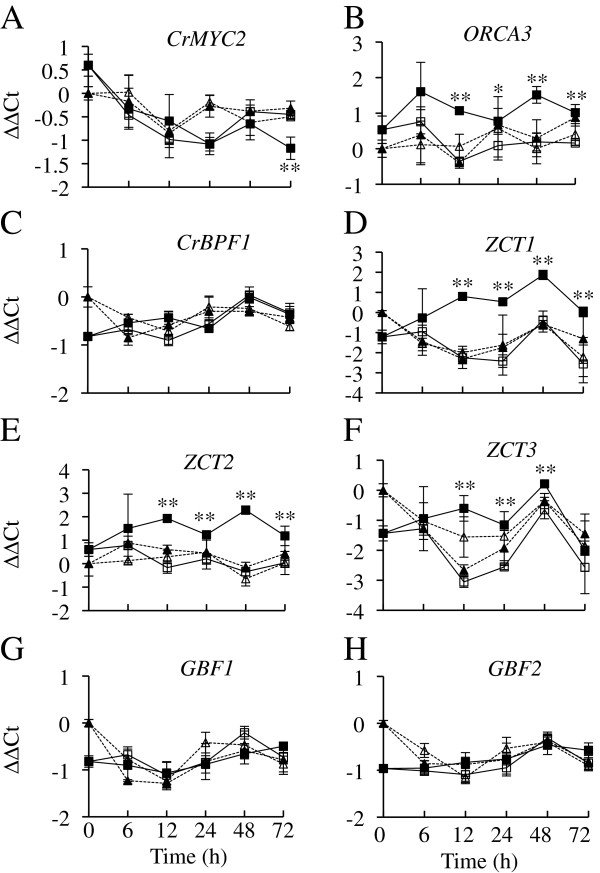
**Time course analysis of TIA regulatory gene expression levels.** Results shown are the: uninduced ORCA2-OE line (□, solid line), 0.02% ethanol-induced ORCA2-OE line (■, solid line), uninduced control line (∆, dashed line) and 0.02% ethanol-induced control line (▲, dashed line). The relative mRNA levels are presented as ∆∆CT. Transcript levels are depicted for the following genes **A)***CrMYC2*, **B)***ORCA3*, **C)***CrBPF1*, **D)***ZCT1*, **E)***ZCT2*, **F)***ZCT3*, **G)***GBF1* and **H)***GBF2*. Results are the average ∆∆CT values of three biological replicates, with two technical replicates for each biological replicate. Error bars indicate standard deviations. Transcript levels of the indicated gene in the induced versus uninduced ORCA2-OE cultures differed at the same time point with: * = p ≤ 0.05, ** = p ≤ 0.01 according to Student’s t-test. Student’s t-test results for the induced versus uninduced cultures of the control line are not depicted.

TIA production appears to be regulated via a complex process. A number of transcriptional activators and repressors have been identified [4-9], including the transcriptional activators ORCA2, ORCA3, CrBPF1, CrMYC1, CrMYC2, CrWRKY1 and CrWRKY2 and the transcriptional repressors ZCT1, ZCT2, ZCT3, GBF1 and GBF2. To determine if ORCA2 regulates TIA transcriptional regulators in addition to TIA biosynthetic genes, transcript levels of *ORCA3, CrBPF1, CrMYC2, ZCT1, ZCT2, ZCT3, GBF1* and *GBF2* were tracked for a 72-h time period in ethanol-induced and uninduced ORCA2-OE and control hairy root cultures. The results of these assays indicate that overexpression of *ORCA2* leads to increased transcript levels of the TIA transcriptional activator *ORCA3* in the induced versus uninduced ORCA2-OE cultures (Figure 9). *ORCA3* transcript levels in the induced ORCA2-OE cultures increase significantly within 12 h of the start of induction and remain elevated throughout the 72-h time course point, compared with the uninduced ORCA2-OE cultures. Overexpression of *ORCA2* had minimal effects on *CrMYC2* transcript levels, with the only significant difference between the ORCA2-OE induced versus uninduced cultures being a very modest decrease in *CrMYC2* transcript levels at the 72-h time point. Interestingly, overexpression of *ORCA2* also causes increases in the steady-state mRNA levels of the TIA transcriptional repressors *ZCT1, ZCT2* and *ZCT3*. The transcript levels of these three zinc finger genes are significantly higher in the induced versus uninduced ORCA2-OE cultures within 12 h of the start of induction and remain elevated through the 72-h time point for *ZCT1* and *ZCT2* and through the 48-h time point for *ZCT3*. In contrast, overexpression of *ORCA2* has no significant effects on transcript levels of *CrBPF1, GBF1* or *GBF2*.

## Discussion

### ORCA2 is a key regulator of the TIA pathway

TIA biosynthesis in *C. roseus* is a complex, multi-step process that is regulated by at least twelve transcriptional regulators. The roles of most of these transcriptional regulators, particularly with regards to the downstream branches of the TIA pathway, remain to be determined [4-9]. As the downstream branches of the TIA pathway are responsible for production of several pharmaceutically important compounds, this lack of information limits attempts to develop models for regulation of TIA production. Therefore, the effects of overexpressing *ORCA2* on transcript levels of 22 TIA biosynthetic and regulatory genes were characterized. The results of the research described here indicate that *ORCA2* overexpression in *C. roseus* hairy roots significantly affects the transcript levels of several key genes from both pathways leading to production of TIA precursors, as well as six of the seven genes characterized from the TIA pathway (Table 1). Overexpression of *ORCA2* causes increased expression of both genes (*ASα* and *TDC*) characterized from the indole pathway. Of the four genes from the monoterpenoid pathway that were characterized, overexpression of *ORCA2* causes increased expression of *G10H* and *LAMT*, but does not significantly affect transcript levels of *DXS* or *CPR*. Perhaps most interestingly, overexpression of *ORCA2* leads to significantly altered transcript levels for almost all of the genes from the TIA pathway that were characterized, including four out of five genes characterized from the downstream branches of the TIA pathway (Table 1). Overexpression of *ORCA2* results in significantly increased transcript levels for *STR*, which encodes the enzyme that catalyzes the first step in TIA biosynthesis. Transcript levels of *T16H*, *D4H* and *PRX1* also increase in response to *ORCA2* overexpression. In contrast, transcript levels of *16OMT* are not significantly affected by *ORCA2* overexpression. Somewhat surprisingly, transcript levels of *SGD* and *DAT* decrease, rather than increase, in response to *ORCA2* overexpression. The effects of *ORCA2* overexpression on *DAT* transcript levels are particularly striking, as *DAT* transcript levels are more than 100 times lower in the ORCA2-OE line 12 h after ethanol induction than in the control line at the zero time point and 12 times lower than in the ORCA2-OE line at the zero time point. These findings that overexpression of *ORCA2* causes significant alterations in transcript levels for key genes from both feeder pathways, the gene that encodes the enzyme that catalyzes the combination of the products of those feeder pathways to form the first TIA, the gene that encodes the enzyme that catalyzes the conversion of the first TIA and four out of five genes characterized from the downstream branches of the TIA pathway indicates that ORCA2 plays a critical role in regulation of TIA metabolism.

**Table 1:** **Effects of *ORCA2 *overexpression on regulation of TIA and TIA-related biosynthetic genes and transcriptional regulators**

**Transgene**	**Result**
*ASα*	+
*TDC*	+
*DXS*	=
*CPR*	=
*G10H*	+
*LAMT*	+
*STR*	+
*SGD*	-
*T16H*	+
*16OMT*	=
*D4H*	+
*DAT*	-
*PRX1*	+
*ZCT1*	+
*ZCT2*	+
*ZCT3*	+
*GBF1*	=
*GBF2*	=
*ORCA2*	+
*ORCA3*	+
*CrBPF1*	=
*CrMYC2*	-

“+” = up-regulation (transcript levels of indicated gene were higher at one or more time points when *ORCA2* was overexpressed); “-” = down-regulation (transcript levels of indicated gene were lower at one or more time points when *ORCA2* was overexpressed). “=” = no change (transcript levels of indicated gene were not significantly different when *ORCA2* was overexpressed).

### *ORCA2* overexpression affects the levels of TIA and related metabolites

Overexpression of *ORCA2* also affects the levels of multiple TIAs and related metabolites (Table 2). In characterizing the indole pathway, tryptophan levels were found to be below detection limits and tryptamine levels were found to increase within 24 h in response to *ORCA2* overexpression. Higher tryptamine levels are consistent with the increased *ASα* and *TDC* transcript levels caused by *ORCA2* overexpression. *ASα* encodes the alpha subunit of anthranilate synthase, which catalyzes the first committed step in the tryptophan biosynthetic pathway and is feedback-inhibited by tryptophan [36]. The concurrent increase in *TDC* expression (Figure 5), however, increases the metabolic flux from tryptophan to tryptamine thus reducing the feedback inhibition of anthranilate synthase by tryptophan. In contrast, of the two metabolites from the terpenoid pathway that were characterized, loganin levels are not significantly affected and secologanin levels are reduced at both the 24- and 72-h time points in response to *ORCA2* overexpression. Two factors may account for the reduced secologanin concentration in response to *ORCA2* overexpression. One factor is that *ORCA2* overexpression has a smaller effect on the genes in the terpenoid pathway (Figure 6) than on the genes in the indole pathway (Figure 5). The other factor is that the increase in *STR* expression (Figure 7) may result in more rapid use of secologanin to form strictosidine.

**Table 2:** **Effects of *ORCA2 *overexpression on the levels of selected metabolites in the TIA and feeder pathways**

**Transgene**	**Result**
Tryptophan	BD
Tryptamine	+
Loganin	=
Secologanin	-
Strictosidine	-
Ajmalicine	+
Serpentine	+
Catharanthine	+
Lochnericine	=
Hörhammericine	-
Tabersonine	-
16-hydroxytabersonine	+
19-hydroxytabersonine	+
Vindoline	BD
Vinblastine	BD
Vincristine	BD

“+” = level of indicated metabolite was higher at one or more time points when *ORCA2* was overexpressed; “-” = level of indicated metabolite was lower at one or more time points when *ORCA2* was overexpressed; “=” indicates no significant change in metabolite level when *ORCA2* was overexpressed; “BD” indicates the metabolite was below detection levels.

The transgenic hairy root samples were also analyzed for the levels of 13 TIAs or TIA-related compounds. Three of these TIAs, vindoline, vinblastine and vincristine, were present at levels below the detection threshold. The levels of strictosidine, the first TIA produced by combining the products of the indole and terpenoid pathways, are significantly lower within 24 h of the beginning of *ORCA2* overexpression. This result was somewhat unexpected as the gene (*STR*) that encodes the enzyme that catalyzes the formation of strictosidine is induced by *ORCA2* overexpression and the gene (*SGD*) that encodes the enzyme that converts strictosidine to cathenamine/4,21-dehydrogeissoschizine/epicathenamine [12] is inhibited by *ORCA2* overexpression. However, decreased strictosidine levels might be explained by the increased expression of genes encoding enzymes for downstream steps, in the TIA pathway, such as *T16H* and *PRX1*, causing an increased rate of metabolite flux to the downstream part of the pathway, in combination with the possibility of *SGD* not being rate limiting for strictosidine metabolism. Alternatively, the reduced *SGD* transcript levels caused by *ORCA2* overexpression may not lead to similar decreases in SGD activity levels. Consistent with the possibilities that SGD activity levels are either not rate limiting or are not decreased, despite the decrease in *SGD* transcript levels, are the findings that ajmalicine and serpentine are transiently increased in response to *ORCA2* overexpression. Both ajmalicine and serpentine, which are made via the same branch of the TIA pathway, are present in significantly higher concentrations 48 h after the start of *ORCA2* induction, but are not present at significantly altered levels at the other time points assayed. Also consistent with these possibilities are findings that *ORCA2* overexpression leads to transient increases in the levels of catharanthine, which is made via a different branch of the TIA pathway, as catharanthine levels are significantly increased 48 h after the start of *ORCA2* induction. The levels of tabersonine, which is used as the starting material for multiple branches of the TIA pathway (Figure 1), are decreased in response to *ORCA2* overexpression. Decreased tabersonine levels in response to *ORCA2* overexpression could be the result of a decreased rate of tabersonine synthesis or an increased rate of tabersonine metabolism. Consistent with the first possibility is the finding that *SGD* transcript levels decrease in response to *ORCA2* overexpression, as SGD activity is necessary for tabersonine production. However, the decrease in *SGD* transcript levels first occurs at a later time point than the decrease in tabersonine levels, making the extent to which decreases in *SGD* transcript levels are responsible for the decreased tabersonine levels unclear. Increases in *T16H* transcript levels in response to *ORCA2* overexpression are consistent with the possibility that decreased tabersonine levels are due to a higher rate of tabersonine metabolism, as T16H catalyzes the first step for one branch of the TIA pathway, namely the conversion of tabersonine to 16-hydroxytabersonine. However, *T16H* transcript levels first increase at a later time point than the decrease in tabersonine levels, making the extent to which increases in *T16H* transcript levels cause decreased tabersonine levels unclear. 16OMT catalyzes the conversion of 16-hydroxytabersonine to 16-methoxytabersonine. *16OMT* transcript levels are not significantly affected by *ORCA2* overexpression. As 16-hydroxytabersonine levels increase within 48 h of the start of *ORCA2* overexpression, these results suggest that 16OMT activity levels may be rate limiting in hairy roots where *ORCA2* is overexpressed. Interestingly, the increase in 16-hydroxytabersonine levels accounts for only a small portion of the substantial decrease in tabersonine levels, raising the possibility that the levels of other tabersonine-related metabolites are raised in response to *ORCA2* overexpression. Consistent with this possibility is the finding that the levels of 19OHTab, which is also made from tabersonine, increase significantly in response to induction of *ORCA2* overexpression. In addition, the identification of a tabersonine-like compound, here designated Unk54, that is present at significantly higher levels in ORCA2-OE induced versus uninduced cultures is also consistent with the idea that the levels of tabersonine-related metabolites increase in response to *ORCA2* overexpression. Lochnericine and hörhammericine are also made from tabersonine, via a different branch of the TIA pathway. Overexpression of *ORCA2* has no significant effects on lochnericine levels, but leads to decreased hörhammericine levels at the 24- and 72-h time points, suggesting that overexpression of *ORCA2* causes an overall decrease in metabolic flux to this branch of the TIA pathway.

### ORCA2 regulates other TIA transcriptional regulators

As ORCA2 is believed to act as a transcriptional activator [18], findings that overexpression of *ORCA2* causes significant decreases in *SGD* and *DAT* transcript levels are somewhat surprising and suggest that regulation of the TIA pathway is complex. A possible explanation for these results is that overexpression of *ORCA2* may cause increased expression of one or more TIA repressors. To examine this possibility and to gain an increased understanding of TIA metabolic regulation, the transcript levels of eight TIA regulatory genes, including all five of the known TIA repressor genes, were examined (Figure 9). *ORCA2* overexpression causes increased transcript levels of *ORCA3*. Interestingly, although overexpression of *ORCA2* causes increased *ORCA3* transcript levels, overexpression of *ORCA3* has no significant affects on *ORCA2* transcript levels [27]. These results suggest that, directly or indirectly, ORCA2 regulates *ORCA3* at the steady-state mRNA level but that ORCA3 does not similarly regulate *ORCA2*. ORCA2 also appears to, directly or indirectly, induce its own expression. Hairy root cultures with higher levels of *ORCA2* transgene expression also have higher transcript levels from the endogenous *ORCA2* gene. In contrast, *ORCA2* overexpression has little effect on *CrMYC2* transcript levels and no significant effects on *CrBPF1* transcript levels.

In addition to regulating some of the TIA transcriptional activator genes, ORCA2 regulates the TIA transcriptional repressor genes *ZCT1*, *ZCT2* and *ZCT3*, but not *GBF1* or *GBF2*. These findings provide a possible explanation for how overexpression of the *ORCA2* transcriptional activator gene may lead indirectly to decreased *SGD* and *DAT* transcript levels by causing overexpression of the ZCT1, ZCT2 and ZCT3 TIA transcriptional repressors, one or more of which may then turn down expression of *SGD* and *DAT*. If this model is correct, one might also expect overexpression of *ORCA2* to lead eventually to a decrease in expression of *TDC* and *STR,* as the ZCTs have been shown to repress expression of both these genes [19]. Although expression of *TDC* and *STR* did decrease between the 48-h and 72-h time points, these decreases were very slight. These results may be due to the competing effects of ORCA2 induction and ZCT repression on expression of *TDC* and *STR*. In addition, differences in the half-lives of the transcripts produced by different genes will cause differences in the timing with which decreases in gene transcript levels are observed. Induction of *ZCT1*, *ZCT2* and *ZCT3* expression in response to *ORCA2* overexpression may help explain why most of the observed alterations in TIA metabolite levels in the *ORCA2*-induced cultures are transient. Interestingly, overexpression of *ORCA3* also results in increased transcript levels of specific TIA repressor genes [27].

### Model for ORCA2 regulation of the TIA pathway

**Figure 10:**
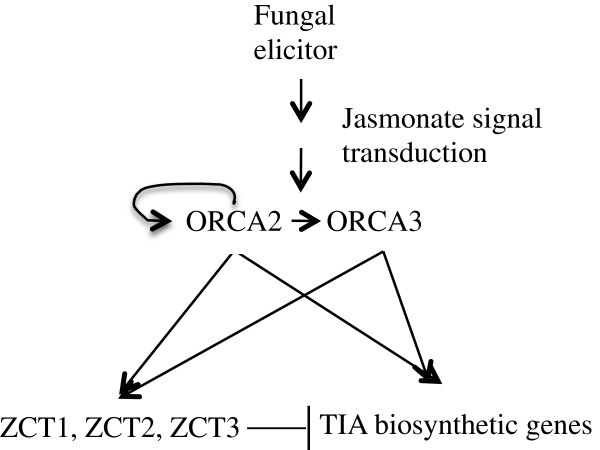
**Model for the role of ORCA2 in regulation of TIA biosynthesis.** A possible model for ORCA2 regulation of TIA biosynthesis is presented. The signal triggered by binding of elicitors activates the jasmonic acid signaling pathway, which then activates ORCA2 and ORCA3. ORCA2 and ORCA3, together with other transcriptional regulators (not shown), induce expression of specific TIA biosynthetic genes. ORCA2 also, directly or indirectly, up-regulates its own expression as well as expression of *ORCA3*. ORCA2 (this work) and ORCA3 [27] also, directly or indirectly, induce expression of the *ZCT1*, *ZCT2* and *ZCT3* TIA transcriptional repressor genes. As the activities of ZCT1, ZCT2 and ZCT3 increase they begin to repress expression of particular TIA biosynthetic genes. As a result, increased expression of ORCA2 or ORCA3 tends to cause increases in TIA biosynthetic gene transcript levels and TIA metabolite levels that are both transient and limited in magnitude.

Based on the results of this study and of other studies, a possible model for regulation of the TIA pathway by ORCA2 has been developed (Figure 10). It should be noted that the available data might also be consistent with other models. In addition, ORCA2 might induce expression of the indicated genes either directly or indirectly. For example, ORCA2 might affect expression of some genes indirectly, such as via effects on ORCA3 expression. In the model presented, *ORCA2* expression is induced via the jasmonate-signaling pathway as a defense mechanism against environmental stresses, such as insect attack or fungal elicitation. Increased expression of *ORCA2*, as well as signaling via the jasmonate-signaling pathway, leads to increased expression of the ORCA3 transcriptional activator. Whether induction of ORCA3 expression via the jasmonate-signaling pathway occurs independently of *ORCA3* induction by ORCA2 is currently unclear. Increased ORCA2 (this study) and ORCA3 [27] activity levels then induce expression of partially overlapping sets of key genes from the TIA pathway and both TIA feeder pathways. The increased expression of the TIA biosynthetic genes in turn affects the levels of several TIAs and related metabolites, which may provide protection against the triggering environmental stress. At approximately the same time that ORCA2 and ORCA3 are inducing expression of TIA biosynthetic genes, they are also (directly or indirectly) inducing expression of the TIA transcriptional repressors ZCT1, ZCT2 and ZCT3. As the levels of these TIA transcriptional repressors rise, they begin to decrease transcription of specific TIA biosynthetic genes. As a result, induction of ORCA2 leads to alterations in the transcript levels of TIA biosynthetic genes and in TIA metabolite levels that are often transient. ORCA3 has been proposed to act in a similar manner [27]. This model for ORCA2 action is partially based on previous findings on the role of ZCTs in repressing activation of *STR* and *TDC* expression by the ORCAs [19], the fact that the ZCTs are transiently induced by fungal elicitors or methyl jasmonate [19,27] and characterization of the effects of *ORCA3* overexpression on expression of TIA regulatory genes [27]. This model describes a mechanism whereby alterations in TIA metabolite levels in response to environmental stresses may be regulated in such a way as to be transient and of limited magnitude.

## Conclusions

Although 12 TIA transcriptional regulators have been identified, very few studies have been done on any of these regulators, with the exception of ORCA3 [7-9,18-24]. Results presented here indicate that ORCA2 plays a critical role in regulation of TIA metabolism. ORCA2 regulates key genes from both feeder pathways, as well as the genes (*STR* and *SGD*) encoding the enzymes that catalyze the first two steps in TIA biosynthesis. ORCA2 may play an especially important role in regulation of the downstream branches of the TIA pathway as it regulates four out of five genes characterized from this part of the pathway. Based on an analysis of the effects of *ORCA2* overexpression on transcript levels of other TIA regulators, a possible model for the mechanism by which ORCA2 helps regulate TIA metabolism has been developed (Figure 10). This model, together with previous work [27], describes a mechanism that allows alterations in TIA metabolite levels in response to external stimuli to be transient and limited in magnitude.

## Methods

### Materials and growth conditions

*Catharanthus roseus,* Vinca Little Bright Eye (http://www.neseed.com), was used for these experiments. Seeds were surface sterilized and germinated in Gamborg’s B5 medium (Sigma, St. Louis, MO, USA) supplemented with Gamborg’s vitamins (Sigma, St. Louis, MO, USA) in the dark at 26°C. After two weeks, seedlings were shifted to a 16-h-light/8-h-dark cycle with a light intensity of 44 μmol m^-2^ s^-1^ and grown for an additional 4 weeks prior to being inoculated with *Agrobacterium tumefaciens*. The genes analyzed in this study are: *16OMT* [GenBank: EF444544], *ASα* [GenBank: AJ250008], *CPR* [GenBank: X69791], *CrBPF1* [GenBank: AJ251686], *CrMYC2* [GenBank: AF283507.2], *D4H* [GenBank: U71605], *DAT* [GenBank: AF053307], *DXS* [GenBank: AJ011840], *EF-1* [GenBank: EU007436], *G10H* [GenBank: AJ251269], *GBF1* [GenBank: AF084971], *GBF2* [GenBank: AF084972], *LAMT* [GenBank: EU057974], *ORCA2* [GenBank: AJ238740], *ORCA3* [GenBank: EU072424], *PRX1* [GenBank: AM236087], *SGD* [GenBank: EU072423], *STR* [GenBank: X53602], *T16H* [GenBank: FJ647194], *TDC* [GenBank: X67662], *UBQ11* [GenBank: EU007433], *ZCT1* [GenBank: AJ632082], *ZCT2* [GenBank: AJ632083], *ZCT3* [GenBank: AJ632084].

### Generation of ORCA2 overexpression construct

The current study employed an ethanol-inducible *alc* gene expression system [34,37], generously provided by Syngenta AG, which has been shown previously to be effective in *C. roseus* hairy roots [32]. Total RNA was isolated from *C. roseus* variety Little Bright Eye. cDNA was generated from this RNA and the full-length coding region of *ORCA2* (AJ872340) was amplified using the KOD Hot Start DNA polymerase (Novagen) and the following oligonucleotides: 5′-ACCTGCAGGTCGACGATGTATCAATCAAATGCCCATAATTC-3′ (contains SalI and PstI restriction endonuclease recognition sequences) and 5′-ACCTGCAGGTCGTCTTATTGAGGACGAAGATGACACG-3′ (contains a PstI restriction endonuclease recognition sequence). The PCR product was digested with SalI and PstI before being cloned into a pACN vector [34] that contains an ethanol-inducible promoter, *AlcA*. The pACN plasmid with the *ORCA2* coding region was then digested into two fragments by HindIII. The fragment carrying the *ORCA2* gene and the *AlcA* promoter was recovered and ligated into the destination vector binSRNACatN [34]. The resulting construct, designated binSRNA-ORCA2 (Figure 2), was used for transformation of *Agrobacterium tumefaciens* GV3101.

### Generation of transgenic hairy roots

To generate transgenic *C. roseus* hairy roots carrying the *ORCA2* coding region under the control of an ethanol-inducible promoter, a mixture of cells containing approximately equal numbers of *Agrobacterium tumefaciens* GV3101 cells transformed with binSRNA-ORCA2 and GV3101 carrying the pPZPROL plasmid were co-transformed into 6-week old *C. roseus* seedlings as previously described [33]. The pPZPROL plasmid carries the *rol ABC* genes, which are sufficient to induce hairy root formation in *C. roseus*[33]. Approximately 4 weeks after inoculation, hairy roots appeared on infection sites. When the hairy roots were about 1 cm in length, roots were excised from the seedlings and transferred onto a solid medium containing 30 g L^-1^ sucrose, 6 g L^-1^ agar, 250 mg L^-1^ cefotaxime, half-strength Gamborg’s B5 salts and full-strength Gamborg’s vitamins (pH 5.8). Hairy root selection started 1 week after roots were transferred to the solid medium. To select hairy roots containing the *ORCA2* gene, 50 mg L^-1^ of kanamycin was used. Root lines showing kanamycin-resistance were further screened by using PCR to amplify the sequence that spans the *AlcA* promoter and the *ORCA2* gene (primers: 5′-GGTACTGTCCGCACGGGATGTCCG-3′ and 5′-TTATTGAGGACGAAGATGACACG-3′). Transgenic hairy root lines showing positive results in both the kanamycin-resistance and PCR amplification tests were transferred to 50 mL of half-strength Gamborg’s B5 liquid solution supplemented with full-strength Gamborg’s vitamins and 30 g L^-1^ sucrose in a 250-mL flask for liquid culture. The flasks were kept on a shaker at 225 rpm in the dark and were sub-cultured every 5 weeks. To generate a control hairy root line, *C. roseus* seedlings were transformed with GV3101 carrying only the pPZPROL construct. A hairy root line that adapted well to liquid media was selected for use as a control line in subsequent experiments.

### Induction of transgene expression and tissue collection

To induce *ORCA2* transgene expression, the liquid media for dark-grown 35-d old ORCA2 transgenic hairy root cultures (each started from five individual hairy roots of 3–4 cm length) were replaced with fresh aliquots of either the same liquid medium or the same liquid medium supplemented with 0.02% ethanol. The hairy roots were returned to the dark and were harvested 0, 6, 12, 24, 48 and 72 h after the start of induction (i.e. replacement of the media). Hairy roots transformed with pPZPROL alone were used as a negative control and were treated the same way as the hairy roots transformed with binSRNA-ORCA2 and pPZPROL. Three independent hairy root cultures were harvested for each transgenic hairy root line, time point and media combination. Upon collection, hairy root samples were immediately flash frozen using liquid nitrogen and then stored at -80°C prior to being used in both the gene expression and metabolite analyses.

### RNA extraction and qRT-PCR analyses

Total RNA was extracted as previously described [38] using the Spectrum Total RNA Isolation Kit (Sigma) with on-column DNase I digestion. cDNAs were synthesized using SuperScript II reverse transcriptase (Invitrogen). *ORCA2* and *CrMYC2* transcript levels were analyzed by qPCR using the SYBR Green JumpStart Taq ReadyMix (Sigma, St. Louis, MO) and a Roche LightCycler 480 II. For analysis of other genes, qPCR assay design was performed using the Roche Universal Probe Library (UPL) technology. Each assay design generated a sequence for the forward primer, reverse primer and amplicon and provided the UPL probe number. Quantitative PCR was performed on an ABI 7900 HT (Applied Biosystems) with a 384-well ABI optical plate using the Homebrew master mix (Biomedical Genomics Center, University of Minnesota) and a Roche Universal Probe (Roche Applied Science). Additional file 1 lists all the primers and probes used for the qPCR reactions. qPCR data were normalized by comparison to *EF1* qPCR results for the experiment depicted in Figure 3. For all other experiments qPCR data were normalized using the geometric average [39] of qPCR results for two control genes, *EF1* and *UBQ11,* which were shown previously to be the two most stably expressed genes of those tested in *C. roseus*[40]. For statements of fold changes, a change of one Ct (i.e. a change of one PCR cycle) was estimated to represent a two-fold change in transcript levels. For the experiment depicted in Figure 4, relative *ORCA2* transgene transcript levels were calculated as ∆∆CT. ∆∆CT = ∆CT_ORCA2-endogenous in uninduced control roots at 0 h_ - ∆CT_other_. ∆CT_ORCA2-endogenous in uninduced control roots at 0 h_ = CT_ORCA2-endogenous in uninduced control roots at 0 h_ – CT_EF1/UBQ11 in uninduced control roots at 0 h_. ∆CT_other_ = CT_ORCA2-transgene_ - CT_EF1/UBQ11_ for the same cDNA sample (e.g. cDNA from an induced culture of the ORCA2-OE line at the 24 h time point). Note that ORCA2 transgene transcript levels were normalized versus ORCA2 endogenous gene transcript levels in the uninduced control line at 0 h rather than against ORCA2 transgene transcript levels in the uninduced control line at 0 h because the control line does not carry the ORCA2 transgene. Relative *ORCA2* endogenous gene transcript levels were calculated as ∆∆CT. ∆∆CT = ∆CT_ORCA2-endogenous in uninduced control roots at 0 h_ - ∆CT_other_. ∆CT_ORCA2-endogenous in uninduced control roots at 0 h_ = CT_ORCA2-endogenous in uninduced control roots at 0 h_ – CT_EF1/UBQ11 in uninduced control roots at 0 h_. ∆CT_other_. = CT_ORCA2-endogenous_ – CT_EF1/UBQ11_ for the same cDNA sample (e.g. cDNA from induced cultures of the ORCA2-OE line at the 12 h time point). For the experiments depicted in Figures 5, 6, 7 and 9, relative transcript levels were also calculated as ∆∆CT. ∆∆CT = ∆CT_uninduced control roots at 0 h_ - ∆CT_other_. ∆CT_uninduced control roots at 0 h_ = CT_indicated gene in uninduced control roots at 0 h_ – CT_EF1/UBQ11 in uninduced control roots at 0 h_. ∆CT_other_ = CT_indicated gene_ – CT_EF1/UBQ11_ for the indicated line at the indicated time point and grown under the indicated conditions (e.g. for induced cultures of ORCA2-OE harvested at the 48 h time point).

### Metabolite extraction and analysis

Frozen hairy root tissue samples, collected as described above, were lyophilized and ground to a powder. As previously described [41], approximately 50 mg of the freeze-dried and ground hairy roots were added to a 50-mL centrifuge tube and extracted with 10 mL of methanol using a sonicating probe (Model VC 130 PB, Sonics & Materials, Inc.) for 10 min while held on ice. The extracts were then centrifuged at 4000 rpm for 12 min at 15°C. The supernatant was removed and the biomass was extracted one more time in the same manner. The combined supernatants were passed through a 0.45 μm nylon filter (25 mm, PJ Cobert), dried using a nitrogen evaporator (Organomation Associates, Inc.), reconstituted in 2 mL of methanol, and passed through a 0.22 μm nylon filter (13 mm, PJ Cobert) for HPLC analysis. Extracts were stored at -25°C.

Twenty microliters of the alkaloid extract was injected into a Phenomenex Luna C18(2) column (250 × 4.6 mm) using three solvent systems. The Waters high performance liquid chromatography system consisted of a 1525 binary pump, a 717plus Autosampler, and a 996 Photo Diode Array (PDA) detector. For the measurement of tryptophan (Sigma, St. Louis, MO) and tryptamine (Sigma, St. Louis, MO), a previously described method was used with UV detection at 218 nm [42]. Quantification was performed by comparison to standard curves. For detection of iridoid glycosides a second solvent system was used. Data extracted at 239 nm were used to quantify loganin (Fluka/Sigma, St. Louis, MO) and secologanin (Fluka/Sigma, St. Louis, MO). The previously described method [41] was adapted from two previously published protocols [43,44]. For 30 min, at a flow rate of 1 mL min^-1^, the mobile phase was linearly ramped from a 90:10 to a 75:25 mixture of 1% formic acid (v/v)/0.25% trichloroacetic acid (w/v):acetonitrile. The ratio was then returned to 90:10 and the column was allowed to re-equilibrate. For the detection of TIAs, a third solvent system was used following a previously described method [41]. Data extracted at 254 nm were used to quantify strictosidine (gift from Dr. O’Connor, John Innes Centre, UK), ajmalicine (Fluka/Sigma, St. Louis, MO), serpentine (Sigma-Aldrich, St. Louis, MO), catharanthine (Qventas, Branford, CT), vindoline (ChemPacific Corp., Baltimore, MD), vinblastine (Sigma, St. Louis, MO) and vincristine (Sigma, St. Louis, MO) using standard curves. Data extracted at 329 nm were used to quantify tabersonine (in-house standard), hörhammericine (in-house standard), lochnericine (in-house standard), 19OHTab (in-house standard), 16OHTab (in-house standard), and Unk54 using retention time standards, photodiode array detection, and a comparison to a tabersonine standard curve [45-49]. In house standards of 19OHTab and 16OHTab were generated from *S. cerevisiae* strains expressing TL19H and T16H genes respectively, and identified using LC-MS and NMR (Additional file 2). Metabolite levels were determined based on peak area, with the exception of loganin, which was quantified based on peak height. The third protocol was adapted from a previously published protocol [42] to use LC-MS compatible solvents. 19OHTab and 16OHTab were verified by LC-MS analysis utilizing an Agilent Technologies 1100 series liquid chromatography (LC) system with a binary pump, a temperature–controlled autosampler and a diode-array detector mated with an Agilent MSD model SL ion trap mass spectrometer (MS). 10 μL of the alkaloid extract was injected into the LC-MS system with a Phenomenex Luna C18(2) column (150 × 2.0 mm). The LC-MS protocol is adapted from the third solvent system of HPLC methods for a smaller diameter of column. The mass spectrometer was operated in positive ion mode and full scan data was collected between 50 and 700 m/z. Operation and analyses were performed with ChemStation software (Agilent Technologies, Santa Clara, CA). The LC-MS analysis was performed at the W. M. Keck Metabolomics Research Laboratory. Unk54 was verified as tabersonine-like using its UV absorbance spectra as collected by the PDA. For quantification, a molecular weight of 352.43 was assumed, the same as the molecular weight of lochnericine, 16OHTab, and 19OHTab.

### Statistical analyses

A two-tailed Student’s t-test was used for data analyses. Significant differences were determined based on comparison of results obtained for the ethanol-induced versus uninduced cultures of the *ORCA2* transgene-containing line at each time point. For transcriptional profiling experiments, (**) was used to represent *p* ≤ 0.01 and (*) was used to represent *p* ≤ 0.05. For metabolite profiling experiments, (**) was used to represent *p* ≤ 0.05 and (*) was used to represent *p* ≤ 0.1.

## Abbreviations

16OMT: 16-hydroxytabersonine-16-*O*-methyltransferase; ASα: Anthranilate synthase α subunit; CPR: Cytochrome P450 reductase; CYP71BJ1: CYP71 cytochrome P450 hydroxylase; D4H: Desacetoxyvindoline 4-hydroxylase; DAT: Deacetylvindoline acetyltransferase; DMAPP: Dimethylallyl pyrophosphate; DXS: 1-deoxy-D-xylulose 5-phosphate synthase; G3P: Glyceraldehyde 3-phosphate; G10H: Geraniol 10-hydroxylase; IPP: Isopentenyl diphosphate; JERE: Jasmonate and elicitor-responsive element; LAMT: Loganic acid *O*-methyltransferase; PRX1: Peroxidase; qPCR: Quantitative polymerase chain reaction; SGD: Strictosidine β-D-glucosidase; SLS: Secologanin synthase; STR: Strictosidine synthase; T6: 7E, tabersonine 6,7-epoxidase; T16H: Tabersonine 16-hydroxylase; TDC: Tryptophan decarboxylase; TIA: Terpenoid indole alkaloid.

## Competing interests

The authors declare that they have no competing interests.

## Authors’ contributions

CYL made the plant transformation constructs, generated and analyzed the hairy roots, prepared tissues and extracts for metabolite analyses and isolated RNA for transcriptional analyses. CYL also drafted the initial version of the manuscript. ALL assisted CYL with most of the experiments. GWS contributed to the metabolite extraction and had primary responsibility for measuring metabolite levels and analyzing the metabolite data. LZ helped revise Figure 1, improved the method used for metabolite analyses and aided in development of metabolite standards. SIG and JVS designed the experiments. SIG substantially revised the manuscript. GWS and JVS were also involved in revising the manuscript. All authors read and approved the manuscript.

## Supplementary Material

Additional file 1 **Primer pairs and probes used for qPCR analyses.** This table provides the sequences of the oligonucleotides used as primers to amplify cDNAs during qPCR analyses. This table also provides the numbers of the probes from the Roche Universal Probe Library used during qPCR analyses. Click here for file

Additional file 2 **Alkaloid identification and quantification.** This document provides information about the generation of the 16OHTab and 19OHTab standards. Additional File 2 also provides information regarding the 16OHTab spectra, UV absorbance properties and MS/MS fragment patterns of the metabolites analyzed in this study, and 16OHTab and 19OHTab MS and MS/MS spectra and 16OHTab NMR spectra. Click here for file
