# Supplementary material for: The ORCA2 transcription factor plays a key role in regulation of the terpenoid indole alkaloid pathway
Source: BMC Plant Biol. 2013 Oct 8;13:155. doi: 10.1186/1471-2229-13-155 (PMC3851283; doi:10.1186/1471-2229-13-155)
Supplement: Additional file 1 — Primer pairs and probes used for qPCR analyses. This table provides the sequences of the oligonucleotides used as primers to amplify cDNAs during qPCR analyses. This table also provides the numbers of the probes from the Roche Universal Probe Library used during qPCR analyses. [file 1471-2229-13-155-S1.docx]

**Additional file 1 Primer pairs and probes used for qPCR analyses.** Probe #: Number of the probe from the Roche Universal Probe Library used to detect the amplicon produced using the indicated primer pair. Note that qPCR analyses of transcripts from the *CrMYC2*, *ORCA2* endogenous (ORCA2en) and *ORCA2* trans (ORCA2tr) genes were performed using SYBR Green and so did not make use of a probe from the Roche Universal Probe Library. In addition, the ORCA2tr_R oligonucleotide binds to a region of the transcript encoded by the binSRNA-ORCA2 plant transformation construct that is coded by sequences present on the vector, rather than on the *ORCA2* gene.

| **Primer name** | **Primer sequence** | **Probe #** |
| --- | --- | --- |
| ORCA2_F | tcaacaacgattttgatttttca | 126 |
| ORCA2_R | tccgaagcataatttggtga |  |
| ORCA2en_F | gggatagtaaattttttctttgagtttttt |  |
| ORCA2en_R | atatcaccttccccatgctcat |  |
| ORCA2tr_F | gatcttcatcgtgtcatcttcgtc |  |
| ORCA2tr_R | caagaccggcaacaggattc |  |
| ORCA3_F | ttccagctcggaattgactt | 6 |
| ORCA3_R | cgaccaatttagaaaatctgcaa |  |
| CrBPF1_F | tggaccgagttttatctgctc | 54 |
| CrBPF1_R | ttcccggtttgcttagactg |  |
| CrMYC2_F | aaaaacaaccaccctgcagc |  |
| CrMYC2_R | aatcggctccccattctca |  |
| ZCT1_F | tctcggaggtcatatgagacg | 58 |
| ZCT1_R | cgcctttgcaacaggtttat |  |
| ZCT2_F | aaaaacccttaattttctccatatttc | 9 |
| ZCT2_R | tctcgtacgcttcatcggta |  |
| ZCT3_F | cgcagcaacacaatattcctt | 143 |
| ZCT3_R | acacttgtagagaagcttaggagga |  |
| GBF1_F | cagagaaagctatgagggcaag | 18 |
| GBF1_R | cacccatcaccttttcagttg |  |
| GBF2_F | agaatctgctcggcgatcta | 96 |
| GBF2_R | cgctgagccaattcatca |  |
| TDC_F | aaaatgttcgaagaatgggttaga | 109 |
| TDC_R | gtttctcggtaccacaatttcg |  |
| DXS_F | gagcactcagcagtgcctta | 125 |
| DXS_R | gcttcccgtaattgcctaaa |  |
| G10H_F | gtacaggaactaattgcgtattgc | 106 |
| G10H_R | cgacgtcaaccgcttctc |  |
| CPR_F | ttgcagtgaggaaggagctt | 67 |
| CPR_R | aatccaaatgggtgcaagaa |  |
| STR_F | ttctatggctttttgaaggttaca | 63 |
| STR_R | catatatgtagcagcagacactcaaa |  |
| D4H_F | tgaactttcatgctgctacactc | 143 |
| D4H_R | ccagcctttgtctcatcaaaa |  |
| DAT_F | cacggtagcagggaaatcag | 142 |
| DAT_R | ctggaaatggcaaagattgg |  |
| EF-1_F | ccgtctcccacttcaggat | 119 |
| EF-1_R | cacgaccaacagggacagta |  |
| UBQ11_F | cgtcaaggctaaaattcagga | 31 |
| UBQ11_R | gaatattgtagtcggccaaggt |  |
| SGD_F | cattggtgaaccgtgctatg | 121 |
| SGD_R | agattgtagagtccagatggaaca |  |
| T16H_F | tgattttaaaggaaattcattcgag | 77 |
| T16H_R | tgcccggacatatccttc |  |
| LAMT_F | gaagcccacccaatgaaa | 9 |
| LAMT_R | ggtagcaagaattttgggagtaac |  |
| 16OMT_F | tggagagtataagttcttgtttgagg | 139 |
| 16OMT_R | cggtaccgcctcctatatca |  |
| PRX1_F | actttgcaacaagggcagac | 31 |
| PRX1_R | gggcacttgtgttgcttgt |  |
| AS alpha_F | aggctaaggagcacatcctg | 151 |
| AS alpha_R | cgttcaaaacgctgacttagg |  |

Caption for supplementary table 1: Supplementary table 1 describes the primers and probes that were used for qPCR analyses.
