# Supplementary material for: The ORCA2 transcription factor plays a key role in regulation of the terpenoid indole alkaloid pathway
Source: BMC Plant Biol. 2013 Oct 8;13:155. doi: 10.1186/1471-2229-13-155 (PMC3851283; doi:10.1186/1471-2229-13-155)
Supplement: Additional file 2 — Alkaloid identification and quantification. This document provides information about the generation of the 16OHTab and 19OHTab standards. Additional File 2 also provides information regarding the 16OHTab spectra, UV absorbance properties and MS/MS fragment patterns of the metabolites analyzed in this study, and 16OHTab and 19OHTab MS and MS/MS spectra and 16OHTab NMR spectra. [file 1471-2229-13-155-S2.docx]

**Additional File 2**

**Alkaloid identification and quantification**

**Methods**

**Generation of 16OHTab and 19OHTab standards**

*Catharanthus roseus* tabersonine 16-hydroxylase (T16H) and tabersonine/lochnericine 19-hydroxylase (TL19H), codon-optimized for expression in *Saccharomyces cerevisiae,* were synthesized (GenScript, Piscataway, NJ) with BamHI and EcoRI restriction sites, cloned into pYeDP60, sequenced, and transformed into *S. cerevisiae* WAT11 cells harboring the integrated *A. thaliana* P450 reductase *ATR1*, thus generating one *S. cerevisiae* cell line with T16H, and a second *S. cerevisiae* line with T19H.

For each recombinant *S. cerevisiae* WAT11 strain, a 100 ml cell culture supplemented with 5 mg tabersonine was grown for 24 h after induction at 30 °C. The cells were removed by centrifugation, and the medium was extracted with 100 mL of ethyl acetate three times and concentrated, then analyzed by LC-MS/MS (Figures S1, S2 and S3).

In addition, a larger volume of *S. cerevisiae* WAT11 cells harboring the T16H construct was grown in order to isolate 16OHTab for NMR analysis. One L of cell culture supplemented with 400 mg tabersonine was grown for 24 h after induction at 30 °C. The cells were removed by centrifugation, and the medium was extracted with 1 L of ethyl acetate three times and concentrated. The enzymatic product, 16-hydroxytabersonine, was purified using a semi-preparative-scale HPLC method. 400 μL of the concentrated extract was injected into a Phenomenex Luna® C18(2) column (250×21.2mm, 15μm). At a flow rate of 10 mL min^-1^, a mobile phase of acetonitrile:100 mM ammonium acetate (pH 7.3) (3:7) was used for the first 10 min. The mobile phase was linearly ramped to 1:9 during the next 5 min and maintained for the next 5 min. The mobile phase ratio was then returned to 3:7 and the column was allowed to re-equilibrate for 25 min. ^1^H NMR, ^13^C NMR, COSY, and ^1^H, ^13^C HSQC spectra were recorded on a Bruker 700 MHz spectrometer (Figures S4, S5 and S6).

**^1^H and ^13^C NMR spectrum description of 16-hydroxytabersonine (16OHTab)**

^1^H NMR (700 MHz, CDCl_3_) δ 8.98 (s,1H), 7.10 (d, J=7.8,1H), 6.38(d, J=2.1, 1H), 6.34 (dd, J=2.1, 7.9,1H), 5.79 (m,1H), 5.72 (d, J=9.9, 1H), 3.89 (s, 3H), 3.87 (d, J=6.22), 3.77 (s,3H), 3.48(dd, J=4.6, 15.9, 1H), 3.24 (d, J=15.8, 1H), 3.06 (t, J=7.6, 1H), 2.72 (s, 1H), 2.55 (dd, J=1.3, 15.2, 1H), 2.40 (d, J=15.1, 1H), 2.09 (s, 1H), 2.03(s, 1H), 1.81 (dd, J=4.3, 12.1, 1H), 1.25 (s, 1H), 1.01(m, 1H), 0.89 (m, 1H), 0.64 (t, J=7.4, 3H); ^13^C NMR (700 MHz, CDCl_3_) δ 7.6 27.2 28.7 41.1 44.5 51.2 51.2 51.2 54.6 70.2 92.5 97.9 107.3 122.3 124.6 130.1 133.3 144.5 156.3 167.0 169.1; *m/z*: 353.2 [M+H]^+^.

**Table S1 UV absorbance properties and MS/MS fragment patterns of all the metabolites in this work.**

| Standard | Source | UV absorbance maxima (nm)^(1)^ | MS/MS fragment pattern | |
| --- | --- | --- | --- | --- |
|  |  |  | Precursor ion [M+H]^+^ (m/z) | Main fragment ion [M+H]^+^ (m/z) |
| tryptophan | Sigma | 218, 278 |  |  |
| tryptamine | Sigma | 218, 278 |  |  |
| loganin | Fluka | 223, 241 |  |  |
| secologanin | Fluka | 222, 240 |  |  |
| strictosidine | Gift from Dr. O’Connor, John Innes Centre | 222 |  |  |
| ajmalicine | Fluka | 225, 279 | 353^(4)^ | 222; 210; 178; 144; 117 |
| serpentine | Aldrich | 248, 305, 363 | 349^(4)^ | 317; 289; 263 |
| catharanthine | Qventas | 225, 281 | 337^(5)^ | 174; 144 |
| vindoline | Chempacific | 216, 251, 304 | 457^(5)^ | 439; 397; 188 |
| vinblastine | Sigma | 216, 266 | 811^(5)^ | 793; 751; 733; 680; 649; 542; 522; 355; 337 |
| vincristine | Sigma | 220, 254, 295 | 825^(5)^ | 807; 765; 747; 723; 705; 687 |
| tabersonine | Extracted from *C. roseus* hairy root | 225, 299, 328 |  |  |
| hörhammericine | Extracted from *C. roseus* hairy root | 225, 298, 325 |  |  |
| lochnericine | Extracted from *C. roseus* hairy root | 225, 298, 327 |  |  |
| 16OHTab | Extracted from *S.* *cerevisiae* WAT11 | 247, 328^(2)^ | 353^(6)^ | 321; 293; 265; 244; 184 |
| 19OHTab | Extracted from *S.* *cerevisiae* WAT11 | 229, 296, 331^(3)^ | 353^(6)^ | 335; 321; 303; 277; 228; 168; 144 |

^(1)^All the UV absorbance maxima data except for 16OHTab and 19OHTab are from Guy Sander’s thesis [[1](#_ENREF_1)].

^(2)(3)^Data are from Gudrun Schroder (1999) [[2](#_ENREF_2)] and Lesley-Ann Giddings (2011) [[3](#_ENREF_3)] respectively, and are confirmed in this work.

^(4)^Data are from Federico Ferreres (2010) [[4](#_ENREF_4)].

^(5)^Data are from Hiu Zhou (2005) [[5](#_ENREF_5)].

^(6)^Data are from this work.


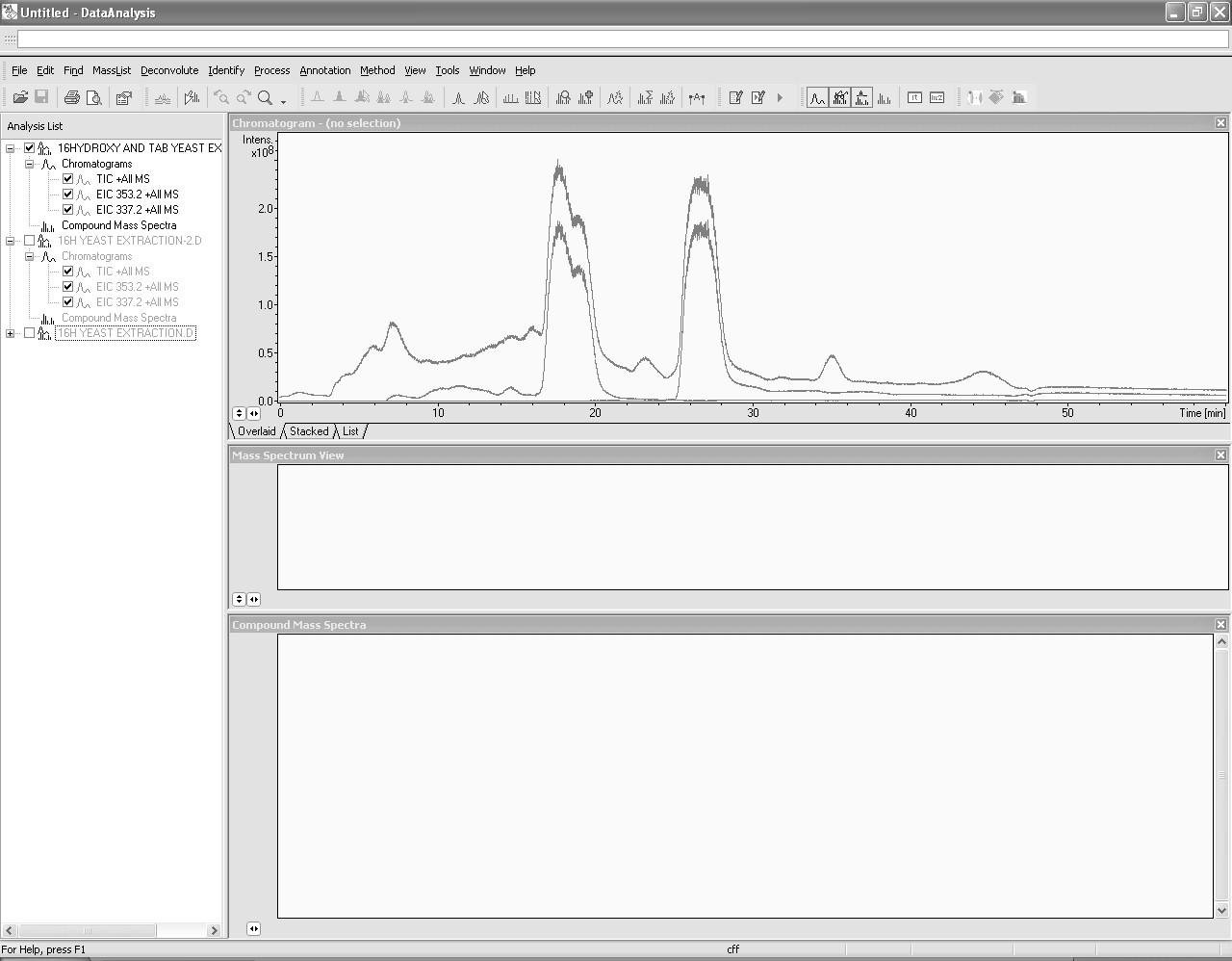


**tabersonine [M+H]^+^ = 337.2**

**16OHTab [M+H]^+^ = 353.2**


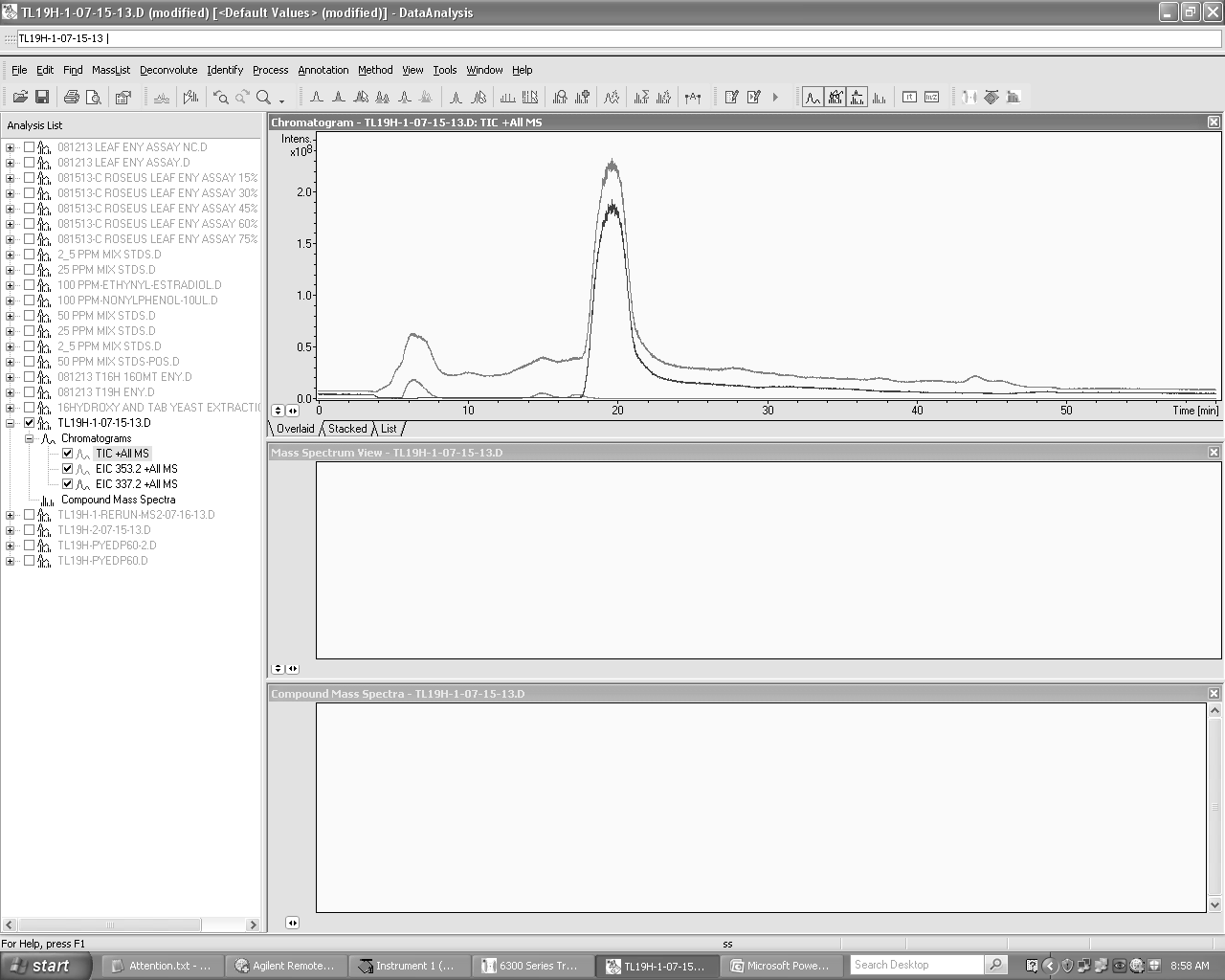


**tabersonine [M+H]^+^ = 337.2**

**19OHTab [M+H]^+^ = 353.2**

**Figure S1 MS spectra of 16OHTab and 19OHTab.**


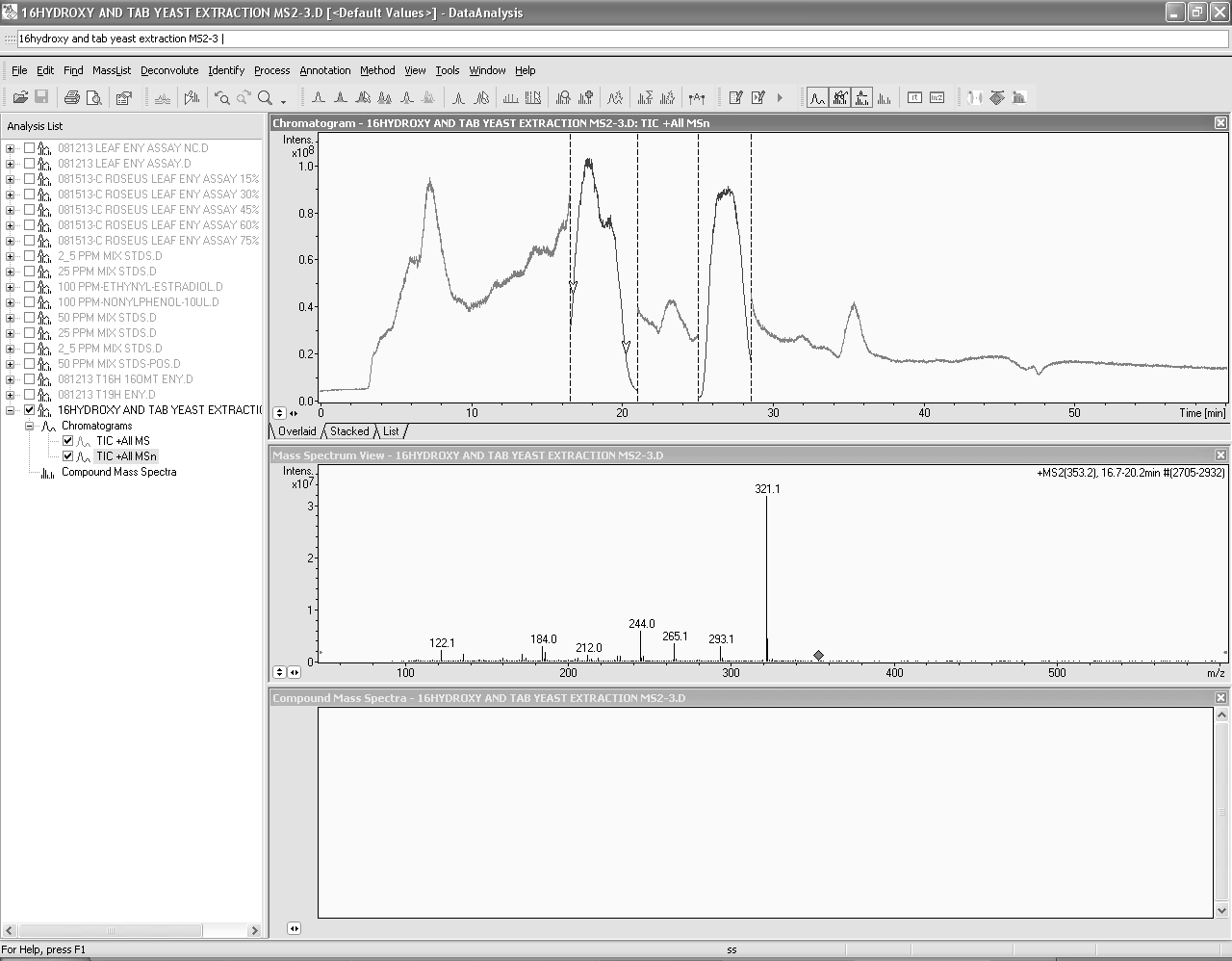


**Figure S2 MS/MS spectra of 16OHTab. ♦**Precursor [M+H]^+^, m/z = 353.2


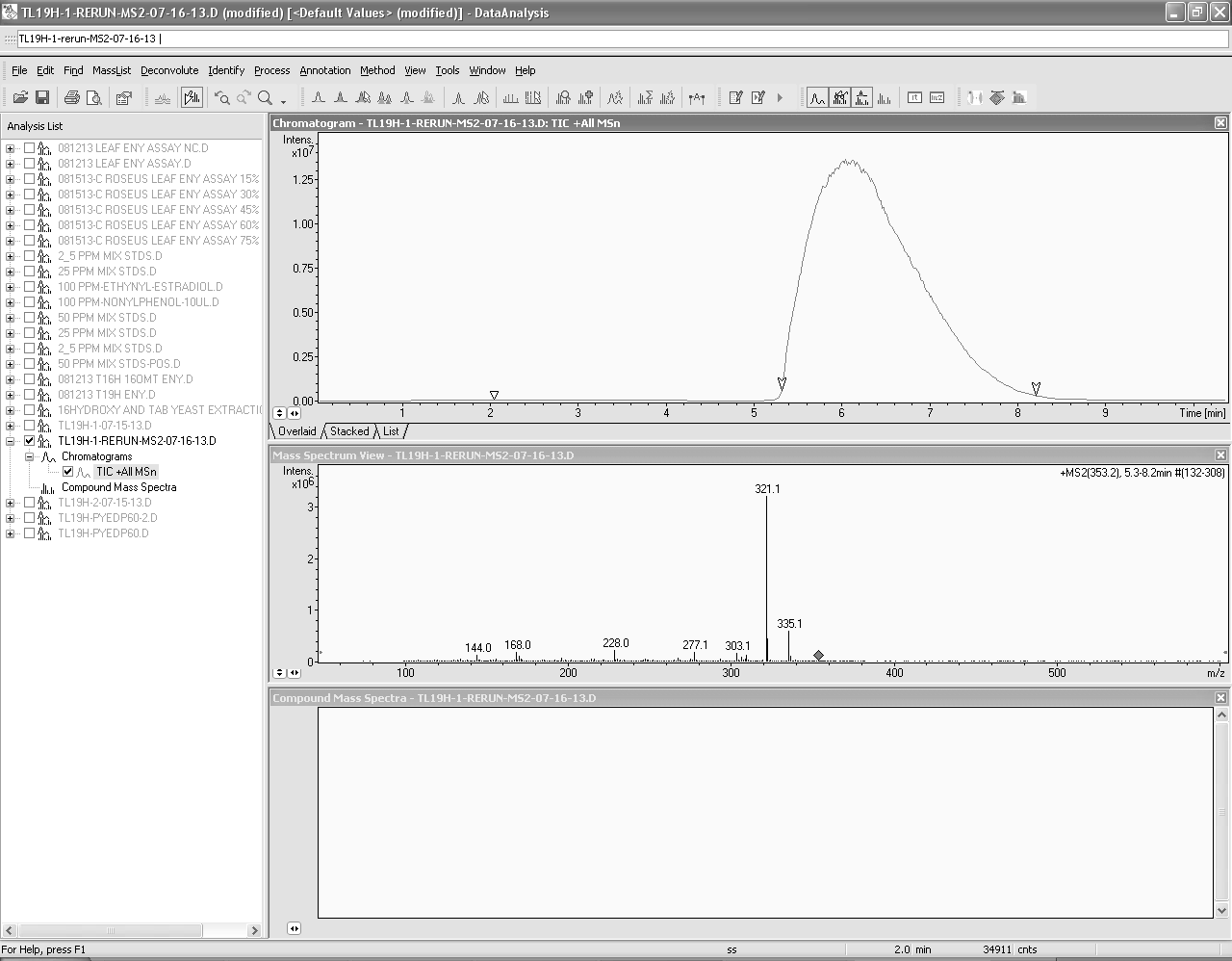


**Figure S3 MS/MS spectra of 19OHTab. ♦**Precursor [M+H]^+^, m/z = 353.2


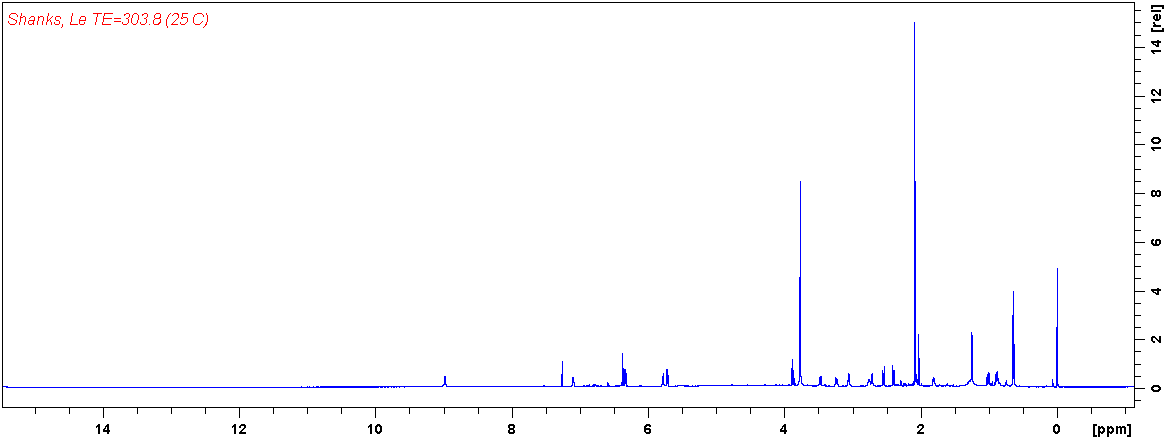


**Figure S4 ^1^H NMR spectrum of 16OHTab.**


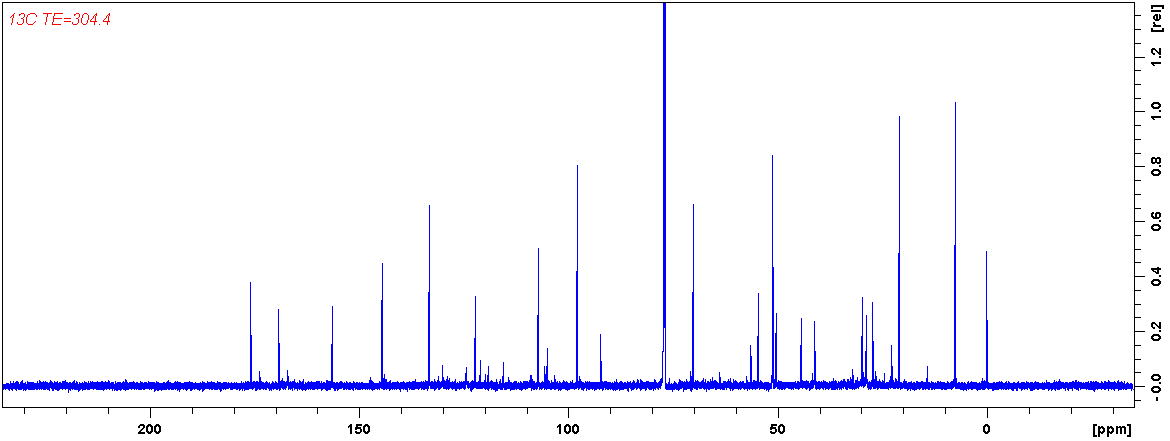


**Figure S5 ^13^C NMR spectrum of 16OHTab.**


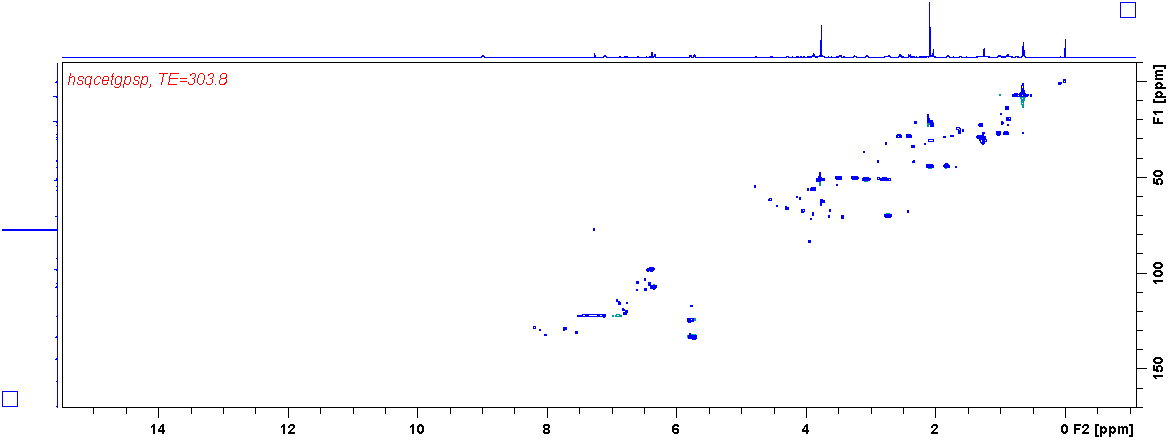


**Figure S6 ^1^H, ^13^C HSQC NMR spectrum of 16OHTab.**

References:

1. Sander GW: **Quantitative analysis of metabolic pathways in *Catharanthus roseus* hairy roots metabolically engineered for terpenoid indole alkaloid overproduction.** *PhD thesis.* Iowa State University, Department of Chemical and Biological Engineering; 2009.

2. Schröder G, Unterbusch E, Kaltenbach M, Schmidt J, Strack D, De Luca V, Schröder J: **Light-induced cytochrome P450-dependent enzyme in indole alkaloid biosynthesis: tabersonine 16-hydroxylase.** *FEBS Lett* 1999, **458:**97-102.

3. Giddings LA, Liscombe DK, Hamilton JP, Childs KL, DellaPenna D, Buell CR, O'Connor SE: **A stereoselective hydroxylation step of alkaloid biosynthesis by a unique cytochrome P450 in *Catharanthus roseus*.** *J Biol Chem* 2011 **28:**16751-16757.

4. Ferreres F, Pereira DM, Valentão P, Oliveira JM, Faria J, Gaspar L, Sottomayor M, Andrade PB: **Simple and reproducible HPLC-DAD-ESI-MS/MS analysis of alkaloids in *Catharanthus roseus* roots***.* *J Pharm Biomed Anal* 2010. **51**:65-69.

5. Zhou H, Tai Y, Sun C, Pan Y: **Rapid identification of vinca alkaloids by direct-injection electrospray ionisation tandem mass spectrometry and confirmation by high-performance liquid chromatography-mass spectrometry**. *Phytochem Anal* 2005 **16:**328-333.

Caption for supplementary file 2: Supplementary file 2 describes alkaloid identification and quantification, as well as generation of 16OHTab and 19OHTab standards.
